# Synthesis of a zeolite-a/MOF-5 composite for the defluoridation of groundwater†

**DOI:** 10.1039/d5ra01995h

**Published:** 2025-05-09

**Authors:** Tessema Derbe, Taju Sani, Enyew Amare Zereffa

**Affiliations:** a Department of Industrial Chemistry, Addis Ababa Science and Technology University P.O. Box 16417 Addis Ababa Ethiopia benyamderbe@gmail.com +251-9-12-97-33-96; b Nanotechnology Center of Excellence, Addis Ababa Science and Technology University P.O. Box 1647 Addis Ababa Ethiopia; c Department of Chemistry, Wachemo University P.O. Box 667 Hossana Ethiopia; d Department of Applied Chemistry, School of Applied Natural Science, Adama Science and Technology University P.O. Box 1888 Adama Ethiopia

## Abstract

Consumption of excessive F^−^ from groundwater harms human health and can cause bone and dental fluorosis. To reduce the excessive F^−^ concentration from groundwater, a novel zeolite-A/MOF-5 (Z-A/MOF-5) composite was synthesized through the solvothermal method. The phase structure, functional group, weight loss, morphology, and elemental composition were characterized by using PXRD, FT-IR, TGA, SEM, and EDX, respectively. The surface charge of the Z-A/MOF-5 composite showed a positive surface up to a pH value of 8.1, which is accessible for the defluoridation of groundwater. The defluoridation efficiency of the Z-A/MOF-5 adsorbent was activated by optimizing defluoridation conditions. The maximum defluoridation efficiency (88.20%) and capacity (11.025 mg g^−1^) were recorded at a pH of 3, 1.2 g L^−1^ of adsorbent dose, 6 h of contact time, and 10 mg L^−1^ initial concentration of F^−^ (*C*_o_) at ambient temperature. However, the defluoridation efficiency of the Z-A/MOF-5 composite still maintained its efficiency (85.50%) up to a pH of 7, which is applicable for the defluoridation of groundwater. The defluoridation data were well fitted with the Freundlich isotherm model and pseudo-second-order kinetics, confirming that defluoridation mainly proceeds *via* chemisorption on the heterogeneous surface of the Z-A/MOF-5 composite. The defluoridation performance of the Z-A/MOF-5 composite was tested on real water samples having 12.25 and 8.5 mg L^−1^ F^−^*C*_o_ taken from Ziway and Kenteri towns, Ethiopia, that reduced the concentration of F^−^ to 1.48 and 0.82 mg L^−1^, respectively. Interestingly, the recyclability study showed defluoridation efficiencies of 88.20%, 87.90%, 86.80%, 85.60%, 82.00%, and 70.10% for the 1^st^, 2^nd^, 3^rd^, 4^th^, 5^th^, and 6^th^ runs, respectively. Consequently, the synthesized composite is a promising adsorbent for practical application.

## Abstract 2

To reduce excessive F^−^ concentrations in groundwater, a novel zeolite-A/MOF-5 (Z-A/MOF-5) composite was synthesized through the solvothermal method. The composite's defluoridation performance was tested on real water samples and it showed promise as an adsorbent for practical application.

## Graphical abstract

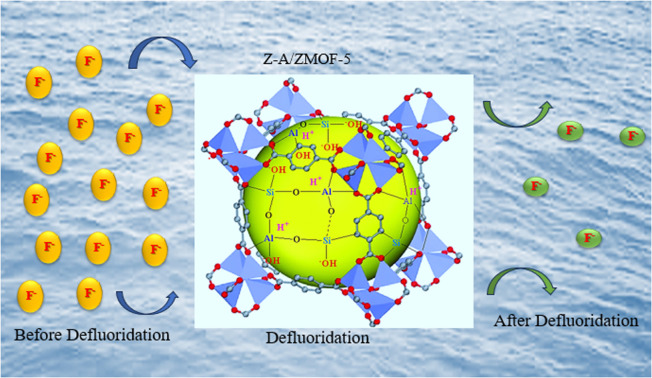

## 1. Introduction

Fluorine is frequently found as fluoride ions (F^−^) due to its high electronegativity and reactivity.^1^ Naturally, F^−^ exists in various sources such as sellaite (MgF_2_), fluorspar (CaF_2_), cryolite (Na_3_AlF_6_), and fluorapatite (Ca_5_(PO_4_)_3_F)).^2–4^ Innumerable anthropogenic activities such as pesticides, dental products, cosmetics, fluoridation processes, and glass factories have significantly raised the F^−^ level in surface and groundwater above the permissible limit.^5,6^ According to the World Health Organization (WHO) guidelines, the tolerable limit of F^−^ in drinking water ranges from 0.5 to 1.5 mg L^−1^.^7^ Within the tolerable limit, F^−^ stimulates the development of teeth, facilitates the mineralization of bone, and prevents the decaying of teeth.^2,8^ However, beyond this threshold limit, F^−^ causes metabolic disorders, reduction of intelligence quotient (IQ) in childhood,^5^ and dental and skeletal fluorosis.^9,10^

This problem is observed in many countries such as Libya, Iran, China, Iraq, South Africa, Kenya, and Ethiopia.^10^ The problem is getting worse in Ethiopia, particularly in the Rift Valley areas such as Awash, Ziway,^11^ Adama, Metehara, and Hawassa.^4^ In the mentioned areas, almost 14 million people are consuming groundwater that contains >1.5 mg L^−1^ F^−^ concentration for drinking purposes.^2,6^ For instance, Ebsa^11^ shows that the Ziway district has F^−^ concentrations from 3.8 mg L^−1^ to 12.7 mg L^−1^. Fito *et al.*^4^ shows that the concentration of F^−^ in the Rift Valley of Ethiopia ranges from 5 to 26 mg L^−1^, which is meaningfully higher than the permissible limit set by the WHO. To reduce excessive F^−^ from groundwater, several removal techniques have been employed, such as chemical precipitation,^1^ membrane separation,^12^ ion exchange,^4^ and adsorption.^13^ Adsorption is the most promising technique for developing countries owing to its cost-effectiveness, ease of operation, low environmental impact, and wider choice of adsorbents.^2,8,12^ This technique has gained substantial attention for the defluoridation of groundwater. In this regard, a plethora of adsorbent materials have been used for the defluoridation of groundwater,^14^ such as zeolite,^15^ activated carbon,^16^ metal oxides,^17^ polymer, and metal–organic frameworks (MOFs).^18^ However, a judicious selection of adsorbent materials is promising for defluoridation purposes. In the present work, zeolite-A (Z-A), MOF-5, and their composite material (Z-A/MOF-5) were tested for the defluoridation of groundwater.

**Fig. 1:**
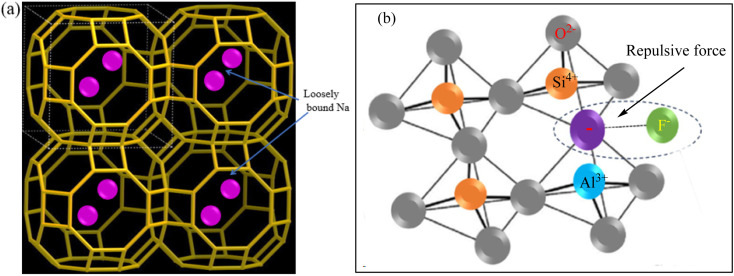
Z-A framework (a) and electrostatic repulsion between zeolite surface charge and F^−^ (b).

Zeolites^2^ are crystalline aluminosilicate consisting of Si^4+^ and Al^3+^ interconnected with four vertex sharing O^2−^ atoms to adopt a tetrahedral structure.^19^ Among various types of zeolites, Z-A [Na_12_(AlO_2_)_12_(SiO_2_)_12_·27H_2_O] (Fig. 1a) has the highest cation exchange capacity owing to its high concentration of Al (Si/Al ∼1).^20^ Thus, Z-A is used for the adsorption of heavy metals *via* cationic exchange processes.^21^ However, Z-A is seldom used for the defluoridation of F^−^ owing to its net negative charge (Fig. 1b) that develops at the Al atom in the Z-A framework.^2,8,17^ Consequently, the defluoridation efficiency of Z-A can be enhanced by compositing Z-A with MOF-5.

**Scheme 1:**
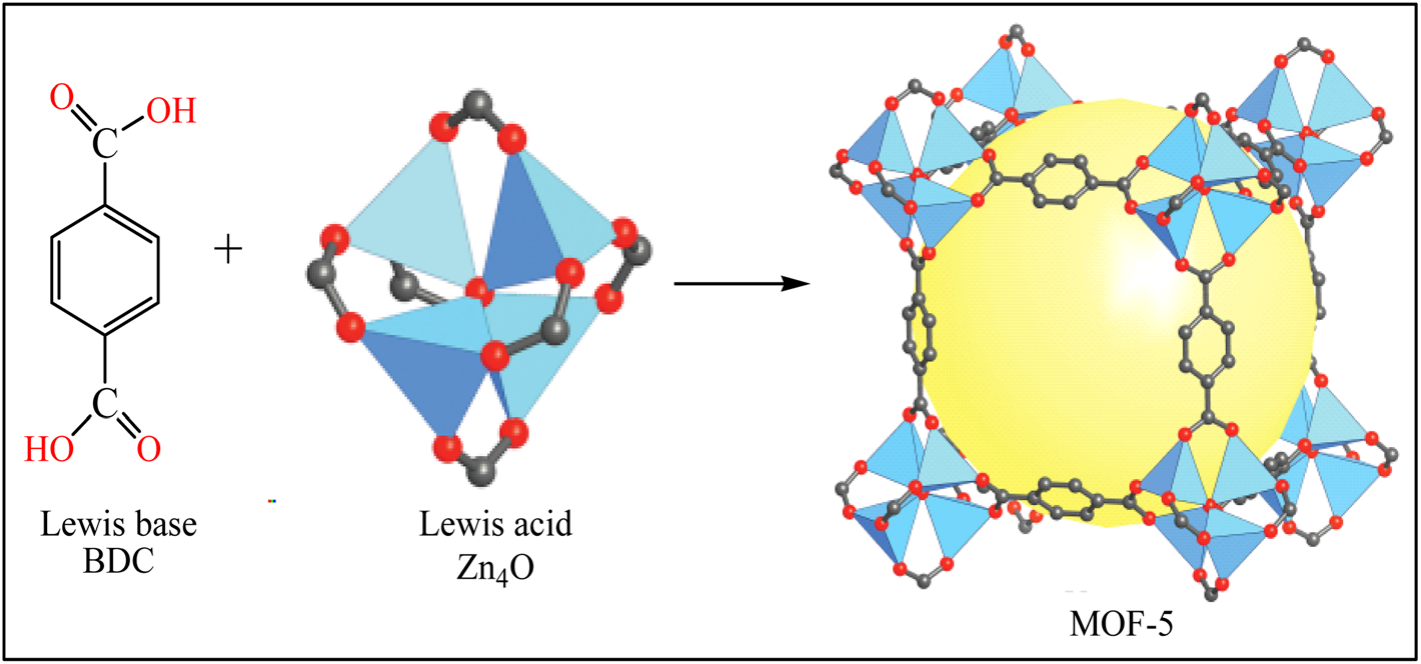
Formation of MOF-5.

The other porous material is a metal–organic framework (MOF). MOFs are an emerging class of organic-inorganic hybrid crystalline that form from positively charged metal ions (Lewis acid) and organic linkers (Lewis base).^22^ Among various MOFs, MOF-5 (Zn_4_O(BDC)_3_) is well-known and consists of a Zn_4_O cluster connected with a 1, 4-benzenedicarboxylate (BDC) organic linker through a coordinated dative bond (Scheme 1).^22,23^ MOF-5 is used for the adsorption of F^−^ owing to its high surface area and surface-rich chemistry.^13,14,24^ Unfortunately, the practical use of pristine MOF-5 for defluoridation ruins substantial objections such as its high production cost, low recovery after defluoridation, and instability in the water environment (hydrolysis and leaching problems).^22^ Consequently, the application of MOF-5 for the defluoridation of F^−^ is scant.

To conquer the existing individual limitations of Z-A and MOF-5, substantial research works have been done on the modification of the adsorbent materials through surface grafting, compositing, incorporating new functional groups, and encapsulating metal ions.^7,25,26^ For instance, Gao and his workers^27^ modified zeolite with zirconia for the treatment of F^−^ from groundwater. Ebsa^11^ modified natural zeolite with cationic surfactant to maximize the defluoridation efficiency from 64.6% to 88.4%. Tabi's research group^17^ modified zeolite with alum for the defluoridation of groundwater. Nevertheless, most of the adsorbents work in acidic media which is a downside for the defluoridation of drinking water.^2,27^ Besides, there are no profound reports on the defluoridation of groundwater using Z-A/MOF-5 composite. Consequently, designing an efficient Z-A/MOF-5 composite that is applicable at a wide pH range (up to 8.1) for the defluoridation of groundwater is paramount. In Z-A/MOF-5 composite, Z-A reduces the production cost and enhances the stability of MOF-5, while MOF-5 boosts the defluoridation efficiency. Therefore, this work was designed to synthesize the Z-A/MOF-5 composite for the defluoridation of groundwater. The defluoridation studies initially optimized various sorption parameters such as pH, adsorbent dose, F^−^*C*_o_, competitor anions, and adsorption time. Besides, the Box–Behnken model was applied using Design Expert 13 software to present the mutual interaction effects of contact time, F^−^*C*_o_, and adsorbent dose on the defluoridation of groundwater.

## 2. Materials and methods

### 2.1. Materials

Zn(CH_3_COO)_2_·2H_2_O (98%), C_2_H_6_O (96%), HCl (37%), NaF (99%) and 1,4-benzenedicarboxylate, BDC (98%) were obtained from Sigma-Aldrich. NaOH (98%), DMF (99%), and CH_3_COOC_2_H_5_ (99%) were from Merck. NaCl (99.5%) and Na_2_SO_4_ (99%) were from Maharashtra, India. Na_3_C_6_H_5_OH.nH_2_O (98%) and Na_2_HPO_4_ (99%) were procured from UDYOG 121001, India. C_2_H_4_O_2_ (99.8%) was from Pentokey Organy, India, while NaNO_3_ (99%) was from Lobe Chemite Ltd. India. All the chemicals and solvents were of analytical grade and were used without further purification. Furthermore, kaolin was collected from Belessa, Hossana zone, Ethiopia, which was used as a natural aluminosilicate source during the synthesis of Z-A. Groundwater sample was collected from Ziway and Kenteri town, Bora Woreda, Ethiopia.

### 2.2. Instruments and equipment

The crystal structures of Z-A, MOF-5, and Z-A/MOF-5 were determined by powder X-ray diffraction analysis (PXRD) using a Philips XRD-1730 with Cu Kα radiation at 40 kV and 35 mÅ with a scanning ranging from 2° to 60°. The surface functional groups were identified using Fourier Transform infrared (FT-IR) spectroscopy (iS50 ABX, USA). The surface morphology and elemental composition of the synthesized adsorbent were characterized using scanning electron microscopy coupled with energy dispersive spectroscopy (SEM–EDX, JEOL JSM-6500F, Japan). The thermal stability of the synthesized adsorbents was studied using thermogravimetric analysis (TGA, DTG-60H, Shimadzu Japan). The concentration of F^−^ was measured using a fluoride ion selective electrode (Metrhom-6.0502.150, Germany). Besides, an orbital shaker (SSL1, UK), digital pH meter (Hanna, UK), ultrasonic bath (SJIA, 950W, China), Teflon lined autoclave, muffle furnace (MSW-251, MAC India), and vacuum drying oven (AOT-DZF-6050, China) were used throughout the work.

### 2.3. Synthesis of adsorbents

#### 2.3.1. Synthesis of MOF-5

**Scheme 2:**
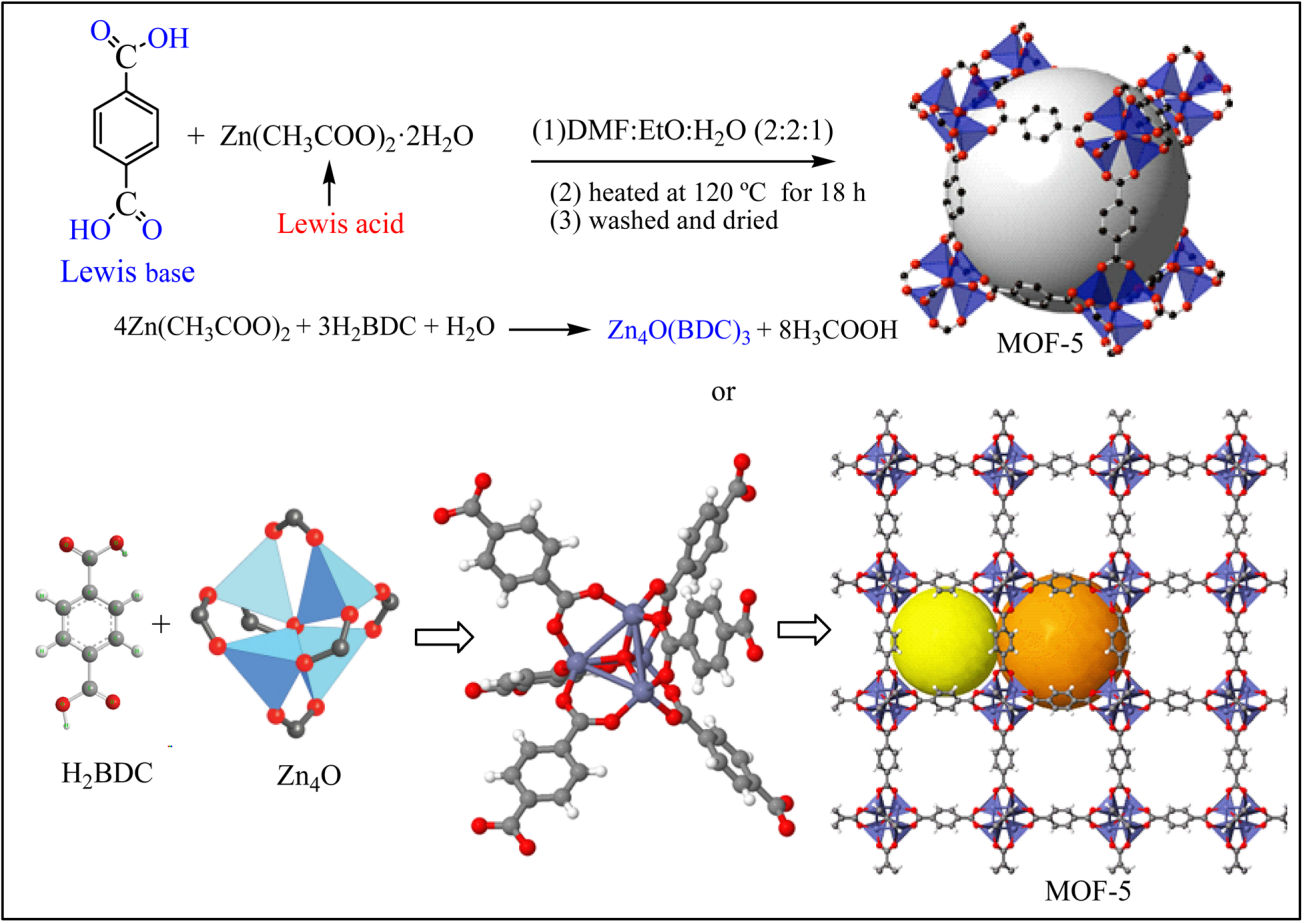
Synthesis of MOF-5 through the solvothermal method.

MOF-5 was synthesized through the solvothermal method^28,29^ using DMF-ethanol-distilled water cosolvents at various Zn(CH_3_COO)_2_·2H_2_O to BDC molar ratios (1 : 1; 1 : 2, 1 : 3, 1 : 4, and 1 : 5). Briefly, a desired amount of BDC and Zn(CH_3_COO)_2_·2H_2_O were dissolved in 50 mL of DMF-ethanol-distilled water cosolvents (2 : 2 : 1 volume ratio) (Scheme 2). The mixture was stirred for 25 min and then sonicated for 30 min at 50 °C to form a homogeneous solution. The homogeneous solution was transferred to a Teflon-lined steel autoclave and heated at 120 °C for 18 h for crystallization. The crystal was dispersed in DMF-methanol for 6 h and then centrifuged at 800 rpm. The product was washed many times with deionized water for the removal of impurities.^30,31^ The resulting white precipitate was dried in a vacuum drying oven at 60 °C for 12 h.

#### 2.3.2. Synthesize of Zeolite-A

**Scheme 3:**
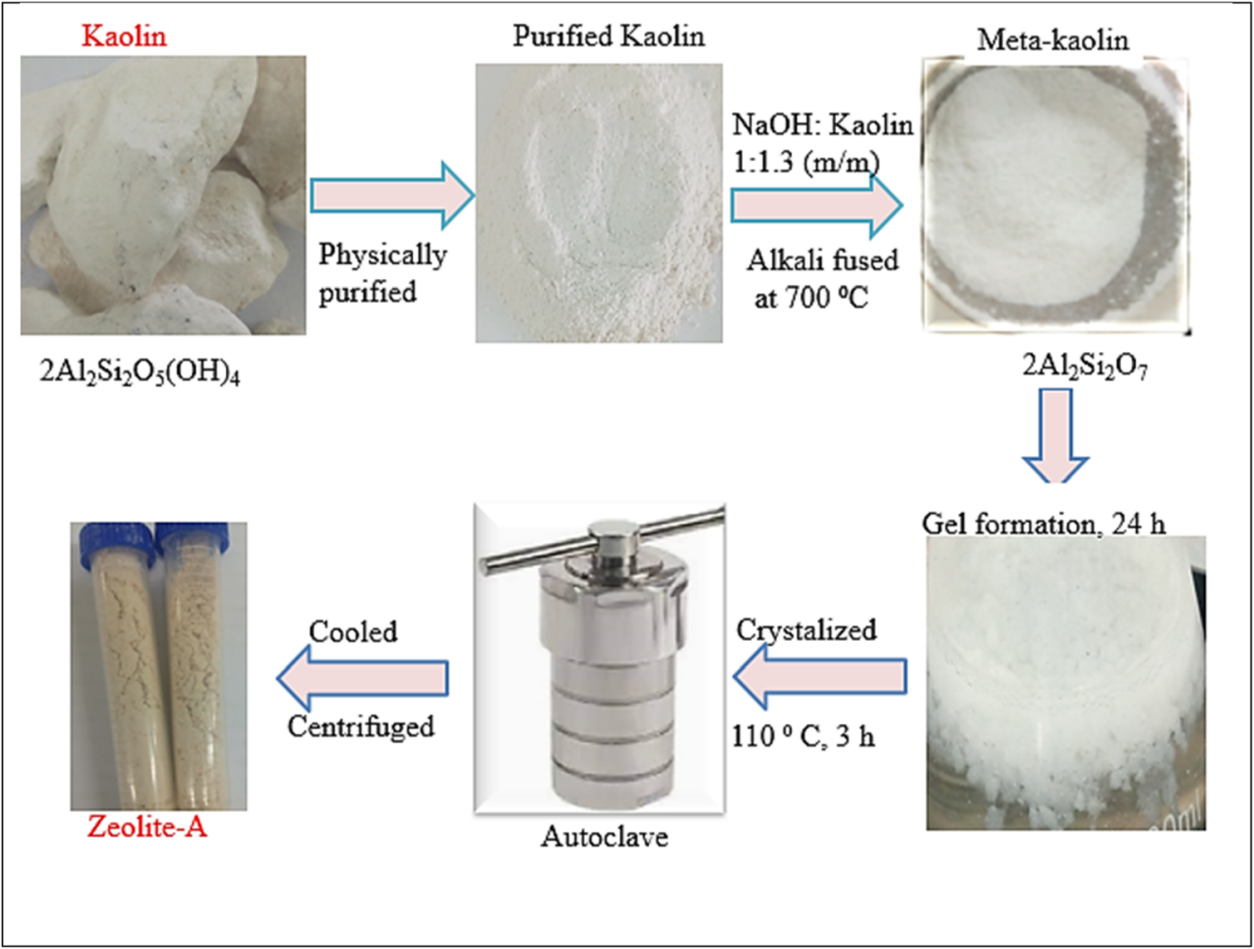
Synthesis of Z-A using the alkali fusion-assisted hydrothermal method.

Z-A was synthesized through alkali fused assisted-hydrothermal method.^19^ Briefly, 40 g of kaolin was soaked with 500 mL of distilled water for 4 days with continuous stirring. The suspension was centrifuged and dried. This physically purified kaolin was mixed with NaOH (1 : 1.3 NaOH to kaolin mass ratio). The solid mixture was ground using a pestle and mortar and incubated for 30 min in a solid-state reaction.^21^ The dry mixed kaolin-NaOH mixture was fused at 700 °C for 1 h. The calcined solid was cooled, ground once again and then sieved through a 150 mm sieve to obtain a fine powder. Two *g* of the fine powder was dispersed in 25 mL of distilled water and heated in a water bath for 1 h at 70 °C for gel formation. The gel was aged for 24 h at the static condition and then crystallized at 110 °C in a Teflon-lined steel autoclave for 3 h. After the autoclave was cooled, the product was washed, centrifuged, and oven-dried at 70 °C (Scheme 3).

#### 2.3.3. Synthesis of Z-A/MOF-5 composite

Z-A/MOF-5 composite was synthesized through solvothermal growth of MOF-5 precursors over a pre-synthesized Z-A surface to reduce competition between Z-A and MOF-5 precursors during nucleation and crystal growth.^26^ 1.0 g of Zn(CH_3_COO)_2_·2H_2_O was dissolved with 15 mL of distilled water–ethanol (2 : 1 v/v). 1.17 g of BDC was also dissolved with 15 mL of DMF in a 100 mL conical flask. Different mass percentages of pre-synthesized Z-A (30, 45, 60, and 75%) were dispersed in four separated 150 mL conical flasks containing 15 mL of DMF. The Zn(CH_3_COO)_2_·2H_2_O solution was added into these dispersed Z-A suspensions and the organic linker solution was added dropwise with continuous stirring.^22,27^ The mixture was sonicated for 15 min at 50 °C and the solution was transferred to an autoclave and heated at 120 °C for 18 h (Fig. S1†). After the autoclave was quenched, the suspensions were centrifuged, washed, and dried in a vacuum drying oven overnight at 60 °C.

### 2.4. Defluoridation studies

#### 2.4.1. Determination of point of zero charge (pH_PZC_)

The zero-point charge of the as-synthesized adsorbents (Z-A, MOF-5, and Z-A/MOF-5) was investigated using the acid–base titration method.^32^ The pH of the 0.5 M NaCl solution was adjusted to 3, 5, 7, 9, and 11 using 0.05 M HCl and 0.05 M NaOH. Synthesized adsorbent (0.05 g) was added into 30 mL of each of the pre-pH adjusted solution. The solution was shaken for 90 min using an orbital shaker (SSL1, UK) and equilibrated for 24 h. Hereafter, the solution was centrifuged, and the pH values of each filtrate were measured using a pH meter (Hanna, UK). The pH_PZC_ of the adsorbents was identified from a common intersection point of the curve of initial pH and their corresponding ΔpH change.^33^

#### 2.4.2. Parameter optimization

Before the defluoridation test, a 1000 ppm stock solution of F^−^ was prepared by dissolving 1.105 g of NaF in 0.5 L deionized water. The desired concentrations of F^−^ solution were prepared for the defluoridation test. Standard solutions (2, 4, 6, 10, and 14 mg L^−1^) were prepared through serial dilution from the stock solution to construct the calibration curve (Fig. S2†). These standard solutions were set based on the mean F^−^ concentration in the Rift Valley of Ethiopia. Then, the defluoridation test was conducted by optimizing experimental parameters such as pH, adsorbent dose, *C*_o_ of F^−^, and contact time. To study the impact of pH on the defluoridation test, batch adsorption was carried out at pH values of 3, 5, 7, 9, and 11 using 0.05 M HCl and 0.05 M NaOH solutions at 0.4 g L^−1^ of adsorbent dose, 10 mg L^−1^ of *C*_o_, and 6 h contact time.^27^ To study the effect of F^−^*C*_o_, working concentrations of 5, 10, 15, 20, 30, and 40 mg L^−1^ were prepared from the stock solution for the subsequent defluoridation tests^4^ (contact time = 6 h, pH = 7, adsorbent dose = 1.2 g L^−1^). The effect of adsorbent dose on F^−^ removal efficiency was studied at adsorbent doses of 0.4, 0.8, 1.2, 1.6, and 2 g L^−1^ (*C*_o_ = 10 mg L^−1^, contact time = 6 h, pH = 7). The effect of contact time on the defluoridation efficiency of F^−^ by the as-synthesized Z-A/MOF-5 composite was studied by varying the adsorption time (3, 6, 9, 12, and 15 h) at a pH of 7, using 1.2 g L^−1^ of adsorbent dose, and 10 mg L^−1^ of *C*_o_. To study the effect of co-existing anions (Cl^−^, NO_3_^−^, SO_4_^2−^, PO_4_^3−^, and CO_3_^2−^) on defluoridation efficiency, a desired amount of sodium salt was dissolved in 30 mL of 10 mg L^−1^ F^−^ to form a 10 mg L^−1^ salt solution.^3^ The mixture was shaken at 160 rpm for 90 min, equilibrated for 6 h, and then filtered. After filtration, 5 mL of TISAB was added to a polyethene bottle containing 20 mL filtrate, and the F^−^ residual was measured using FISE.^17^ A real water sample analysis was also carried out for groundwater containing 12.25 and 8.5 mg L^−1^ F^−^*C*_o_ from Ziway and Kenteri town, Bora Woreda, Ethiopia, respectively. The adsorption capacity (*q*_e_), and the defluoridation efficiency (*R*) of F^−^ were calculated by eqn (1) and (2), respectively.^4,34^1
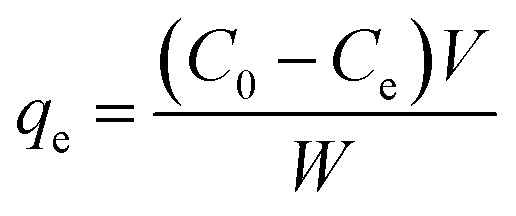
2
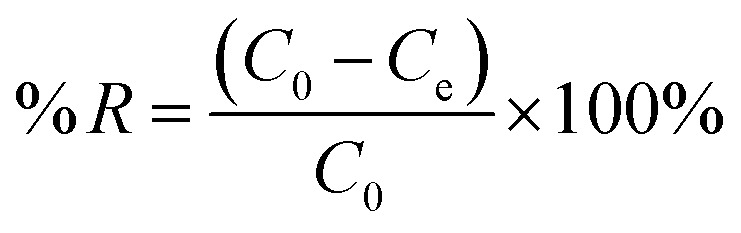
where *C*_0_ and *C*_e_ represent the initial and equilibrium fluoride concentration (mg L^−1^), respectively, *V* (mL) is the volume of solution, and *W* (g) is the amount of the adsorbent.

#### 2.4.3. Adsorption isotherms

The monolayer and multilayer defluoridation mechanism on the adsorbent surface were proposed using Langmuir and Freundlich isotherms.^35^ The Langmuir isotherm model forecasts that the adsorbate sticks into identical surface sites through homogeneous adsorption energy, whereas the Freundlich isotherm predicts that the adsorbate traps on the heterogeneous adsorbent surface. The linear form of Langmuir and Freundlich isothermal models were proved using eqn (3) and (4), respectively.^27^3
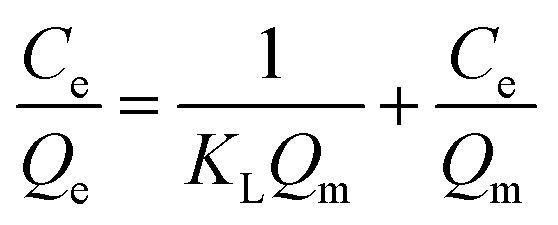
4
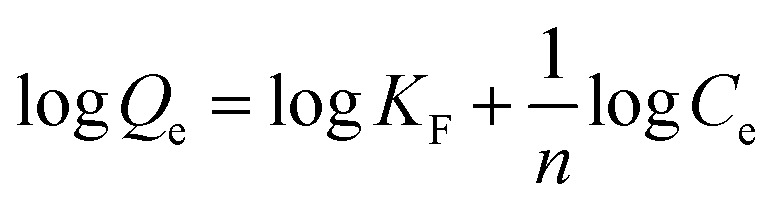
where *C*_e_ is the equilibrium concentration of adsorbate in the solution (mg L^−1^), *Q*_e_ is the amount of adsorbate adsorbed per unit weight of adsorbent (mg g^−1^), *Q*_m_ is the maximum up taking capacity (mg g^−1^), *K*_L_ is the Langmuir constant related to energy (L mg^−1^), *K*_F_ and *n* are dimensionless constants: relative adsorption capacity and intensity of adsorption, respectively.

#### 2.4.4. Adsorption kinetics

The adsorption kinetics of the synthesized adsorbent was studied by varying contact time (3, 6, 9, 12, and 15 h). The adsorption kinetics models (pseudo-first-order, pseudo-second-order, and intraparticle diffusion models) were studied using eqn (5)–(7) to investigate the adsorption mechanism and the rate of F^−^ adsorption on the adsorbents' surface.^1,36^5
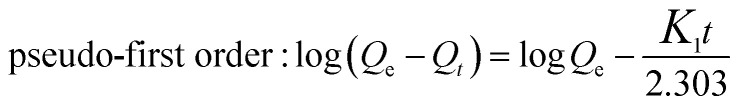
6
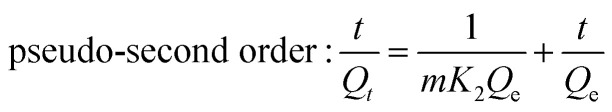

7 intraparticle diffusion: *Q*_*t*_ = *K*_ad_*t*^1/2^ + *C*

where *Q*_e_ is the amount of adsorbate adsorbed per unit weight of adsorbent at equilibrium (mg g^−1^), *Q*_*t*_ is the amount of adsorbate adsorbed per unit weight of adsorbent (mg g^−1^) at time *t* (min), and *k*_1_ and *k*_2_ are the first and second pseudo-order rate constants for the adsorption (min^−1^), respectively. *K*_ad_ (mg g^−1^ min^−1/2^) is the intraparticle diffusion rate constant, and *C* is the intercept, reflecting the boundary layer effect or surface adsorption.

#### 2.4.5. Recyclability test of adsorbents

The F^−^ saturated Z-A/MOF-5 adsorbent was dispersed in 40 mL of 0.01 M NaOH solution and shaken using a shaker at 160 rpm for 90 min for the desorption of F^−^.^10^ The adsorbent was centrifuged and dried in an oven at 60 °C overnight to be reused for five successive defluoridation tests. For each run, the F^−^ concentration of the filtrate was measured using FISE at optimized conditions (*C*_o_ = 10 mg L^−1^, contact time = 6 h, pH = 7, adsorbent dose = 1.2 g L^−1^).

## 3. Results and discussion

### 3.1. Characterization of adsorbent

#### 3.1.1. PXRD analysis

**Fig. 2:**
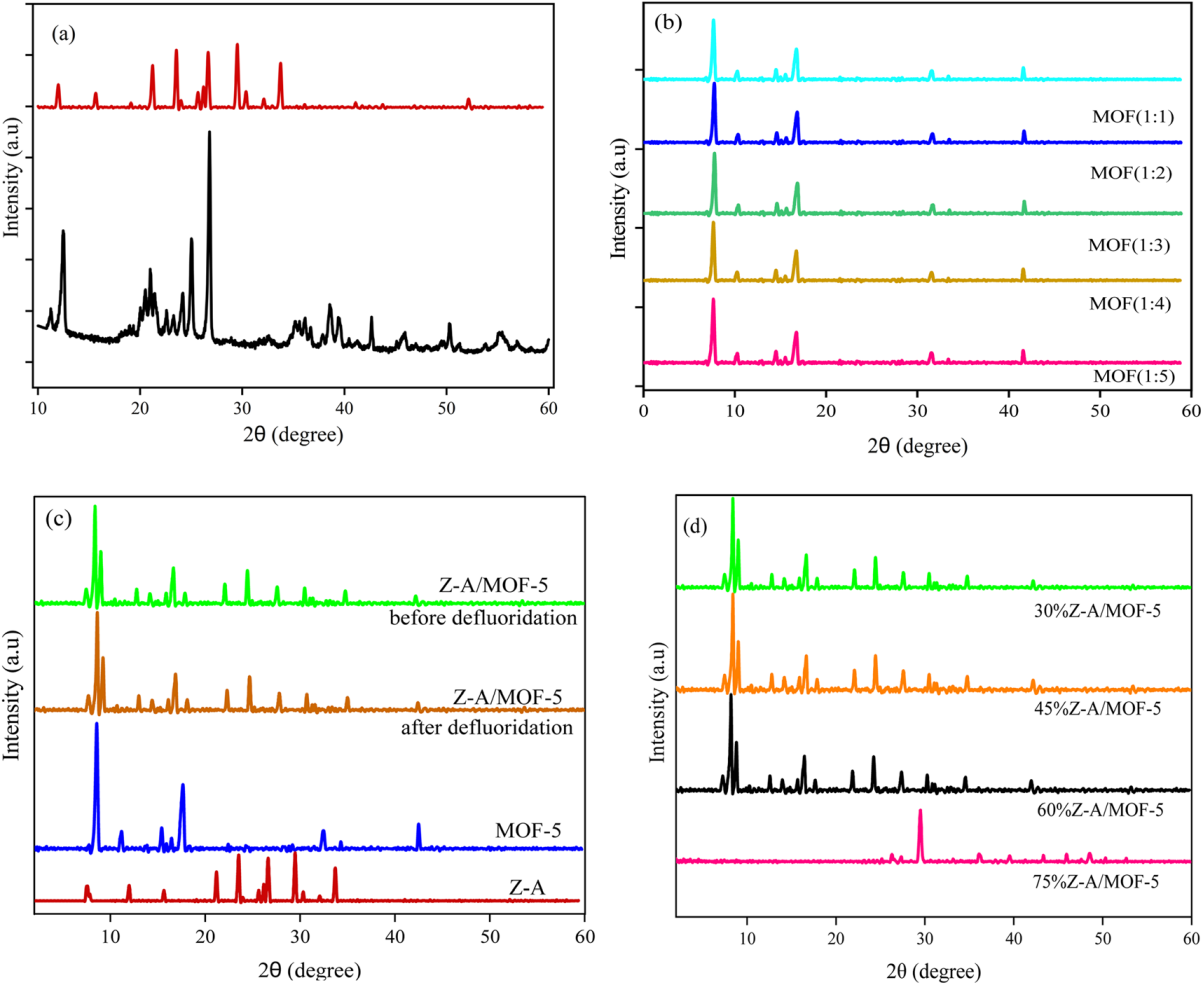
PXRD patterns of (a) raw kaolin and synthesized Z-A, (b) MOF-5 at various Zn(CH_3_COO)_2_·2H_2_O to BDC molar ratios, and (c and d) Z-A/MOF-5 composite.

The PXRD patterns of raw kaolin, Z-A, MOF-5, and Z-A/MOF-5 are represented in Fig. 2a–d. The main PXRD peaks at 2*θ* = 12.40, 24.80, and 26.72° indicated the presence of kaolinite and quartz in the raw kaolin.^17,37^ The PXRD peaks of synthesized Z-A at 2*θ* = 12.04, 15.72, 21.22, 23.65, 29.54, and 33.78° corresponded to the (222), (420), (600), (642), (644), and (664) planes, respectively, which concurred with the data reported by Tran *et al.*^38^ The estimated average particle size of the as-synthesized Z-A was 47.42 nm using Scherer's eqn (8). The as-synthesized Z-A showed approximately 76.48% crystallinity according to eqn (9). The PXRD patterns of the synthesized MOF-5 at various molar ratios of Zn(CH_3_COO)_2_·2H_2_O to BDC were illustrated in Fig. 2b. The presence of peaks at 8.78, 11.46, 15.73, and 17.93° (JCPDS NO 96-432-6738) with the respective Miller plane of (110), (200), (211), and (220), respectively suggested the successful formation of MOF-5.^22^ The appearance of the most prominent peak at 8.89° revealed the crystalline nature of the synthesized MOF-5.^39^ Besides, small PXRD peaks at 21.64, 31.54, 33.51, and 42.16° with JCPDS file NO. 36-1451 were associated with trace ZnO nanoparticles within the MOF-5 framework. A similar result was reported by Liu *et al.*^40^ The mean crystalline sizes of the as-synthesized MOF-5 at Zn(CH_3_COO)_2_·2H_2_O to BDC molar ratios of 1 : 1; 1 : 2, 1 : 3, 1 : 4, and 1 : 5 were 27.14, 25.09, 36.76, 41.95, and 45.64 nm, respectively. Their respective % crystallinity were 80.89, 84.16, 79.09, 76.48, and 66.15% at 1 : 1; 1 : 2, 1 : 3, 1 : 4, and 1 : 5 Zn(CH_3_ COO)_2_·2H_2_O to BDC molar ratios, respectively. Furthermore, the preliminary defluoridation experiment at 1 : 1; 1 : 2, 1 : 3, 1 : 4, and 1 : 5 Zn(CH_3_COO)_2_·2H_2_O to BDC molar ratios resulted in 90.20, 92.00, 88.20, 87.02, and 85.00% defluoridation efficiency, respectively. This further supports the results obtained from the % crystallinity and particle size (Fig. S3†). Henceforth, the molar ratio (1 : 2) of Zn(CH_3_COO)_2_·2H_2_O to BDC was selected for further characterization and adsorption studies.

The PXRD diffraction peaks of Z-A/MOF-5 composite at 2*θ* = 8.89, 14.19, 17.94, 27.67, 30.40, and 35.99° suggested the growth of MOF-5 over the Z-A surface (Fig. 2c and d). The PXRD peaks at 27.67, 30.40, and 35.99° also suggested the presence of Z-A in the Z-A/MOF-5 composite. Consequently, the PXRD peaks of the as-synthesized Z-A and MOF-5 remained intact during the preparation of the Z-A/MOF-5 composite.^41,42^ However, as the amounts of Z-A increase, the typical diffraction PXRD peak of MOF-5 diminishes at 75%Z-A/MOF-5; the peak originated dominantly from Z-A matrix materials (Fig. 2d). The PXRD patterns after defluoridation do not show significant change (Fig. 2c), which confirms the stability of the as-synthesized Z-A/MOF-5 composite.^43^ The average particle sizes of the as-synthesized Z-A/MOF-5 composites containing 30, 45, 60, and 75% of Z-A were 26.77, 33.72, 42.04, and 64.87 nm, respectively. The preliminary defluoridation tests for the Z-A/MOF-5 composites resulted in 88.20, 86.00, 82.80, and 72.00% defluoridation efficiency for 30%Z-A/MOF-5, 45%Z-A/MOF-5, 60%Z-A/MOF-5, and 75%Z-A/MOF-5, respectively (Fig. S3†). The defluoridation efficiency decreased as the amount of host material (Z-A) increased. This suggests that there is active participation of MOF-5 in the defluoridation of F^−^ from groundwater. Consequently, the 30% Z-A/MOF-5 composite was designated for further characterization and adsorption analysis.8
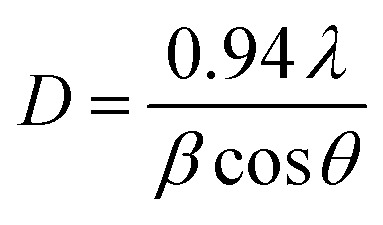
where *D* is the crystallite size in nm, *λ* is the radiation wavelength (0.154 nm), *θ* is the diffraction peak angle, and *β* is the line width of the XRD peak at half-peak intensity ((FWHM)).9
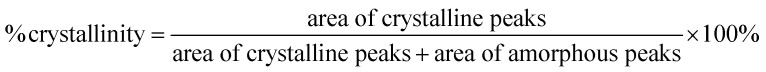


#### 3.1.2. FT-IR analysis

**Fig. 3:**
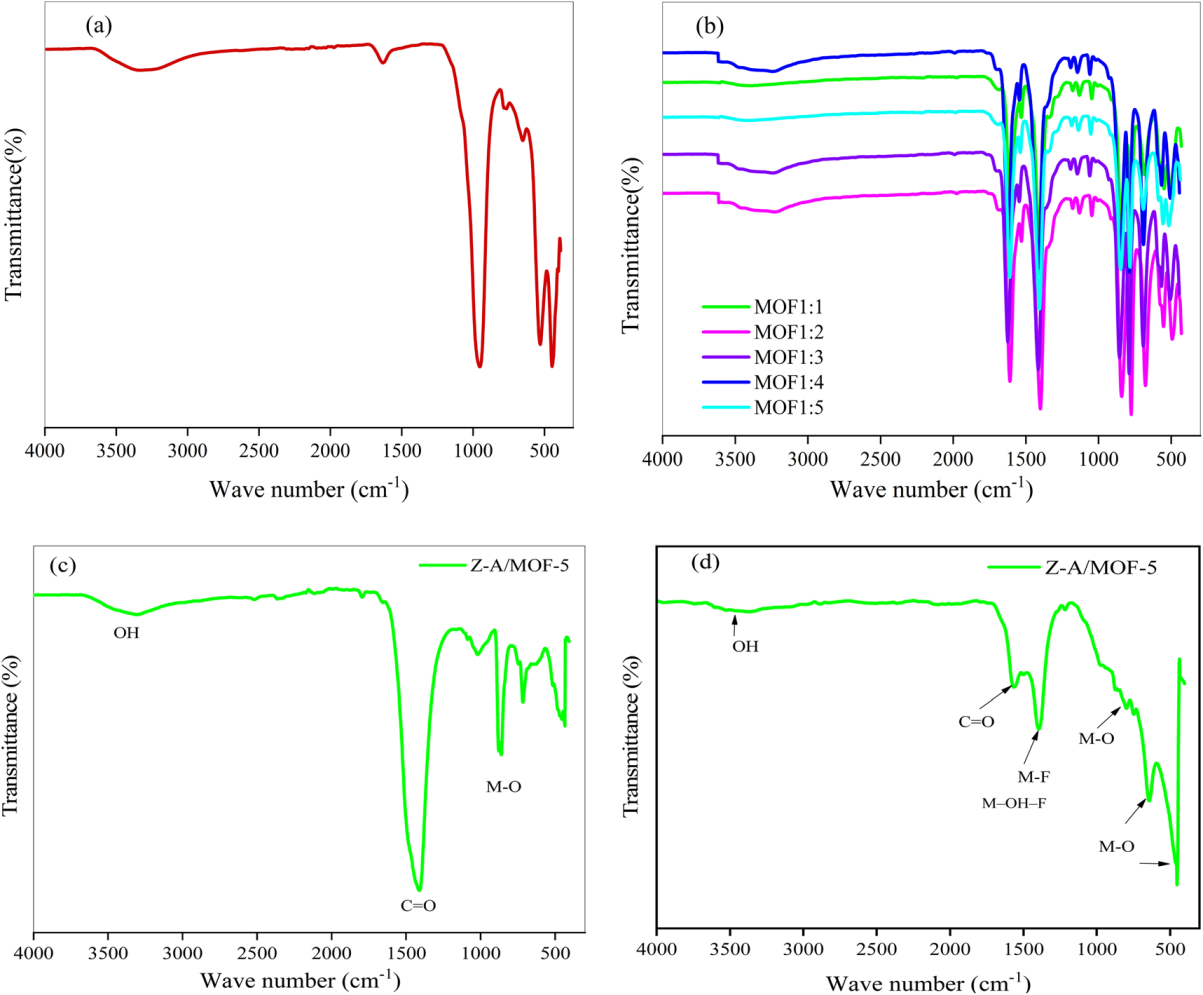
FT-IR spectra of the synthesized (a) Z-A, (b) MOF-5, (c) Z-A/MOF-5 composite before defluoridation, and (d) Z-A/MOF-5 composite after defluoridation.

The FT-IR vibrational bands of the as-synthesized Z-A, MOF-5, and Z-A/MOF-5 are observed between 400–4000 cm^−1^ (Fig. 3a–d). The FT-IR peaks of the as-synthesized Z-A at 968 and 781 cm^−1^ are attributed to the asymmetric and symmetric stretching vibration of MO_4_ tetrahedron (where M = Si or Al), respectively.^17^ The wave numbers at 543 and 457 cm^−1^ are assigned to asymmetric external and symmetric internal vibrations of the double ring of silicon and aluminum tetrahedron.^44^ Peaks at 3400 and 1648 cm^−1^ suggested the stretching and bending vibration of water molecules in the Z-A framework (Na_12_(AlO_2_)_12_(SiO_2_)_12_·27H_2_O), respectively. These FT-IR results agreed with the reported values of Z-A by Bu, *et al.*,^45^ which suggested that Z-A was completely prepared from kaolin.

The FT-IR result of the as-synthesized MOF-5 was illustrated in Fig. 3b. The wave numbers at 1577.06 and 1387.89 cm^−1^ are associated with asymmetric and symmetric stretching vibrations of C=O, which corresponds to the attachment of carboxylate ligand to the Zn_4_O center of the MOF-5 framework.^46,47^ The appearance of peaks at 664, 546, and 446 cm^−1^ further verified the formation of Zn–O bonds in the MOF-5 framework.^42^ Peaks at 843 and 768 cm^−1^ indicate C–H bending of the aromatic out-of-plane.^18,48^ Besides, a peak at 3357 cm^−1^ is attributed to the O–H stretching vibrations of absorbed moisture.

The FT-IR peaks of the Z-A/MOF-5 composite at 3355 and 1409 cm^−1^ are accredited to the asymmetric stretching vibrations of bridging O–H (Zn–OH–Zn) and carboxylate linker (C=O bond), respectively (Fig. 3c). The disappearance of peaks at 1577.06 cm^−1^ and the red shift from 1387.89 cm^−1^ to 1411 cm^−1^ demonstrated a chemical reaction between Z-A and MOF-5 in the Z-A/MOF-5 composite.^42^ The appearance of a new FT-IR peak at 1564 cm^−1^ (Fig. 3d) in the Z-A/MOF-5 composite after defluoridation suggests the adsorption of F^−^.^16^ This could be due to the formation of M–F bonds (M = Al, Si, or Zn), or the formation of intermediate complexes like Al–OH–F and Zn–OH–F on the surface of the Z-A/MOF-5 composite. This phenomenon correlates with the data reported by Wang *et al.*^43^^.^ The attenuation hydroxyl group vibrations peaks (3200–3600 cm^−1^) suggest the importance of hydrogen bonds for the adsorption of F^−^. The attenuation^43^ of the FT-IR peak at 1393 cm^−1^ also implies the binding of F^−^ in the Z-A/MOF-5 composite sites (Fig. 3d).

#### 3.1.3. SEM-EDX analysis

**Fig. 4:**
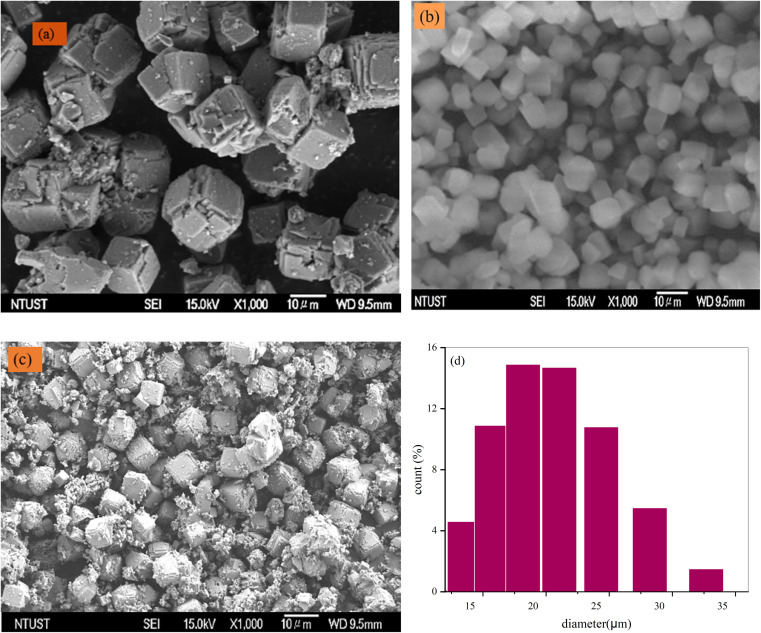
SEM images of the synthesized (a) Z-A, (b) MOF-5, and (c) Z-A/MOF-5 composite and (d) particle size of the Z-A/MOF-5 composite.

**Fig. 5:**
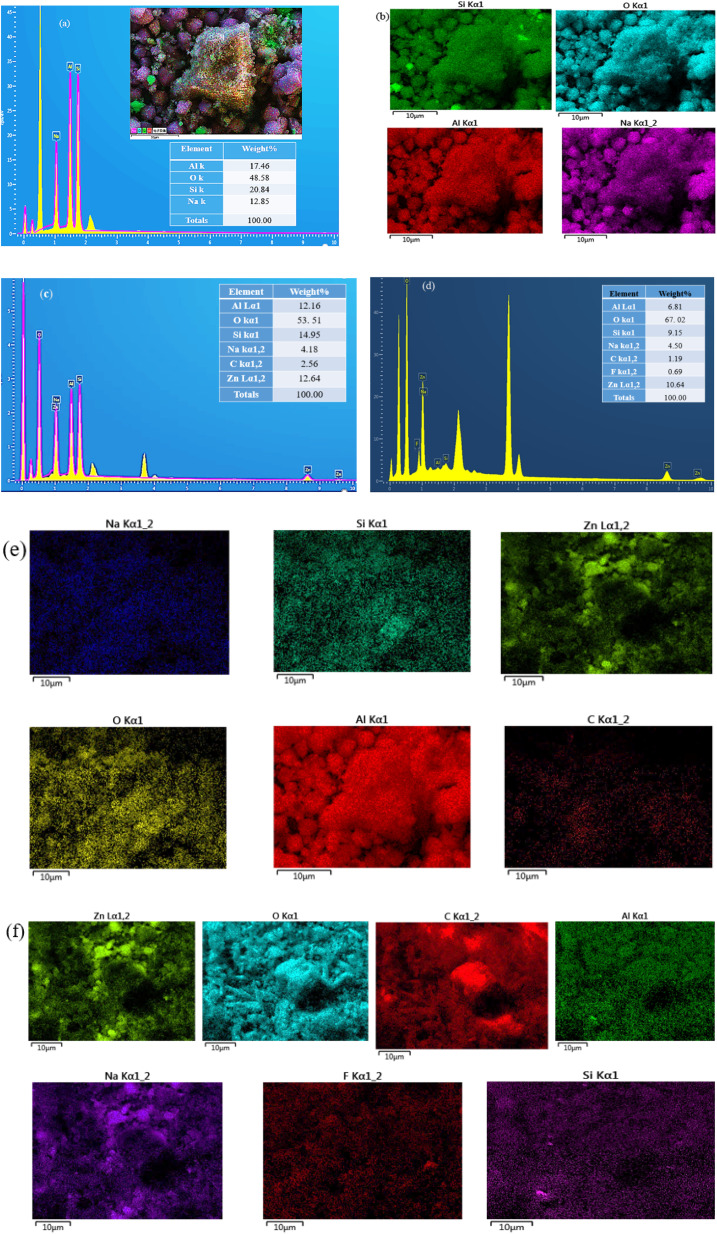
(a and b) EDX images and mapping distributions of Z-A, (c and e) Z-A/MOF-5 before defluoridation, and (d and f) Z-A/MOF-5 after defluoridation.

The SEM-EDX results of the synthesized adsorbents (Z-A, MOF-5, and Z-A/MOF-5) are illustrated in Fig. 4 and 5. The surface morphology of Z-A is a cubic shape (Fig. 4a).^47^ Similar surface morphology was reported by Ayele *et al.*^49^ The average particle size of Z-A was 0.49 μm using ImageJ software. The SEM image of MOF-5 also exhibited a cubic shape with 0.026 μm (Fig. 4b). The cubic morphology of Z-A and MOF-5 is preserved^50^ except for some aggregates with heterogeneous particles in the Z-A/MOF-5 composite (Fig. 4c). This suggests the coexistence of MOF-5 and Z-A in the Z-A/MOF-5 composite. The average particle size of the Z-A/MOF-5 composite changed from 0.49 to 0.18 μm when the MOF-5 grew over the Z-A surface. The estimated diameter of the Z-A/MOF-5 composite from the histogram was 1.021 μm (Fig. 4d), which is consistent with the PXRD result (Fig. 2).

The percentage weight of the elemental composition of Z-A was 20.84% Si, 17.46% Al, 48.58% O, and 12.85% Na, which confirms the purity of the as-synthesized Z-A (Fig. 5). As anticipated, the ratio of Si/Al for the as-synthesized Z-A was 1.19, which is close to 1.^49^ This further supports the formation of Z-A from raw kaolin.^37^ EDX elemental mapping confirms the existence of all anticipated elements in the synthesized Z-A: Na, Al, Si, and O (Fig. 5b). The expected elemental composition of the Z-A/MOF-5 composite was 12.64% Zn, 4.18% Na, 2.56% C, 12.16% Al, 14.95% Si, and 53.51% O (Fig. 5c). The elemental mapping distribution supports the formation of Z-A/MOF-5 composite without any impurity (Fig. 5d). The percentage weight of the elemental composition for the Z-A/MOF-5 composite after defluoridation was 10.64% Zn, 6.81% Al, 67.02% O, 1.19% C, 9.15% Si, 0.69% F, and 4.50% Na which indicates the adsorption of F^−^ on the Z-A/MOF-5 surface (Fig. 5e). The slight decrement of Zn, Al, and Si content after adsorption could be the active participation of those metals in the adsorption of F^−^. Likewise, the ratio of Si/Al before and after defluoridation was 1.23 and 1.34, respectively, which did not show a significant deviation. This suggests the existence of Z-A in the Z-A/MOF-5 composite. Furthermore, the existence of F^−^ in elemental mapping distribution verifies the defluoridation (F^−^ removal) from groundwater (Fig. 5f).

#### 3.1.4. Thermogravimetric analysis

**Fig. 6:**
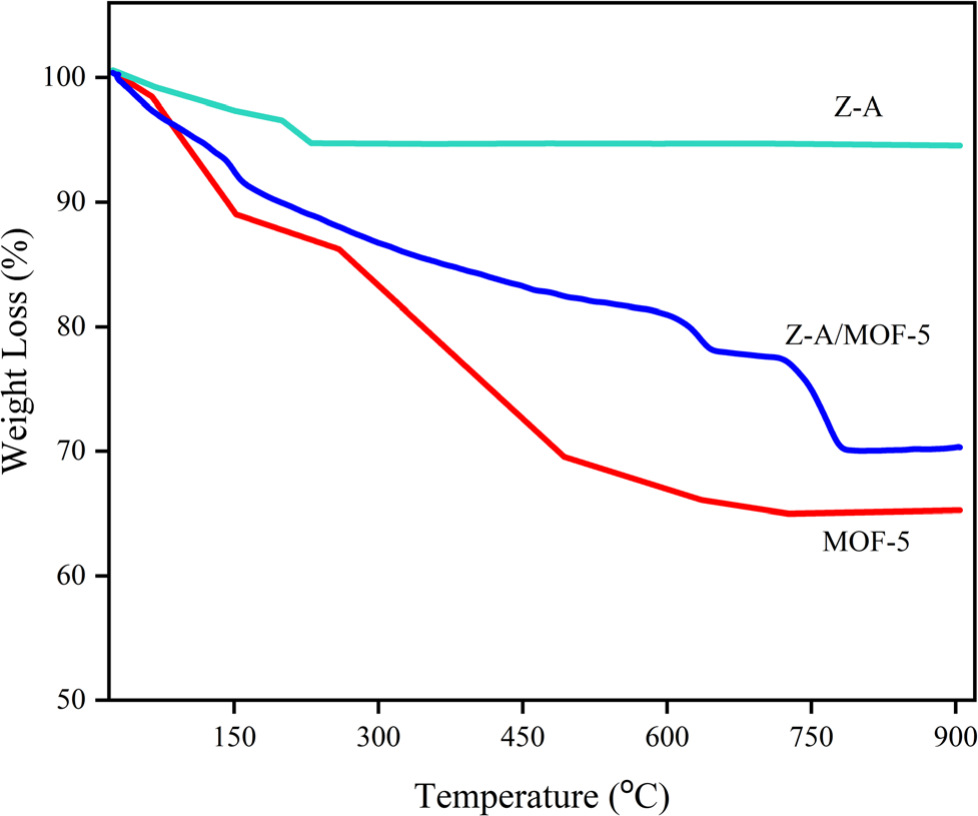
TGA curve of Z-A, MOF-5, and Z-A/MOF-5.

The thermal stability and weight loss of Z-A, MOF-5, and Z-A/MOF-5 were studied using TGA (Fig. 6). The weight loss of Z-A (5.27%) occurred from ambient temperature up to 224 °C. This is due to the evaporation of adsorbed water in the Z-A structure.^26^ The 1^st^ weight loss of MOF-5 is 10.71% (65 to 142 °C) owing to dehydration of the physically adsorbed water molecules.^51^ The 2^nd^ and the 3^rd^ weight losses of MOF-5 are 8.49% (141 to 384 °C) and 8.65% (384 to 470 °C), respectively, which are associated with the obstructed solvent molecules (particularly DMF) and the decomposition of BDC ligand, respectively. The last weight loss from 470 to 524 °C is 7.17%, which corresponds to the decomposition of the MOF-5 framework.^18^ The weight loss of the Z-A/MOF-5 composite is 9.81% (30 to 184 °C), 9.18% (184 to 603 °C), 3.42% (603 to 719 °C), and 7.56% (719 to 745 °C). These correspond to the loss of adsorbed moisture, DMF, and the decomposition of organic linkers in the Z-A/MOF-5 structure, respectively. In a nutshell, the total weight loss of Z-A, MOF-5, and Z-A/MOF-5 was 5.27%, 35.02%, and 29.97%, respectively. Accordingly, Z-A was the most thermally stable, followed by Z-A/MOF-5 and MOF-5.^26^

### 3.2. Defluoridation studies

#### 3.2.1. Point of zero charge (PZC)

**Fig. 7:**
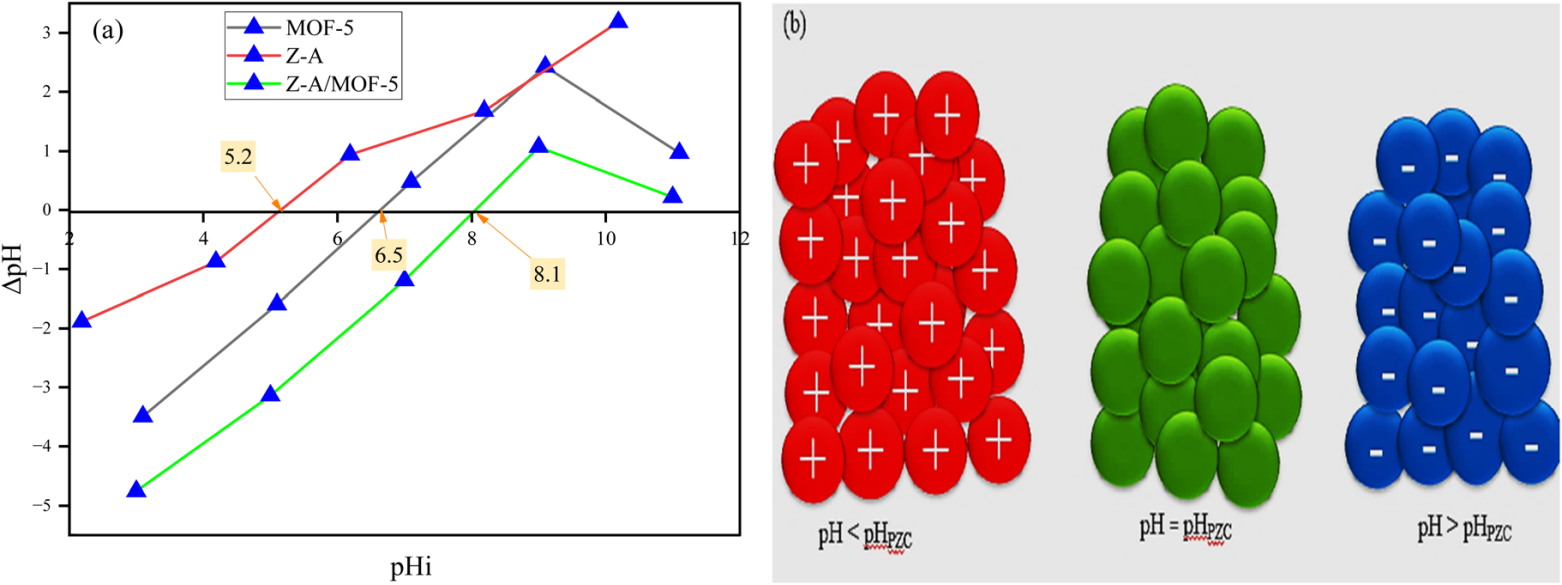
(a) Point of zero charge and (b) surface nature of synthesized adsorbents.

The point of zero charge of the as-synthesized Z-A, MOF-5, and Z-A/MOF-5 is 5.2, 6.5, and 8.1, respectively (Fig. 7). Below PZC, the surface of the as-synthesized adsorbents is protonated and positively charged, which enhances the defluoridation of F^−^.^4^ At pH values beyond PZC, the surface of synthesized adsorbents is deprotonated (HO^−^ is formed), which results in low defluoridation capacity owing to electrostatic repulsion forces between the adsorbents surface and adsorbates.^19,51^ The surface of the Z-A/MOF-5 composite is positive up to a pH of 8.1, which is feasible at a wide pH range (pH 3 to 7) for the defluoridation of F^−^ from groundwater. Interestingly, the as-synthesized Z-A/MOF-5 composite is used at a wide pH range compared to its precursors (Z-A and MOF-5).

#### 3.2.2. Effect of pH and initial concentration

**Fig. 8:**
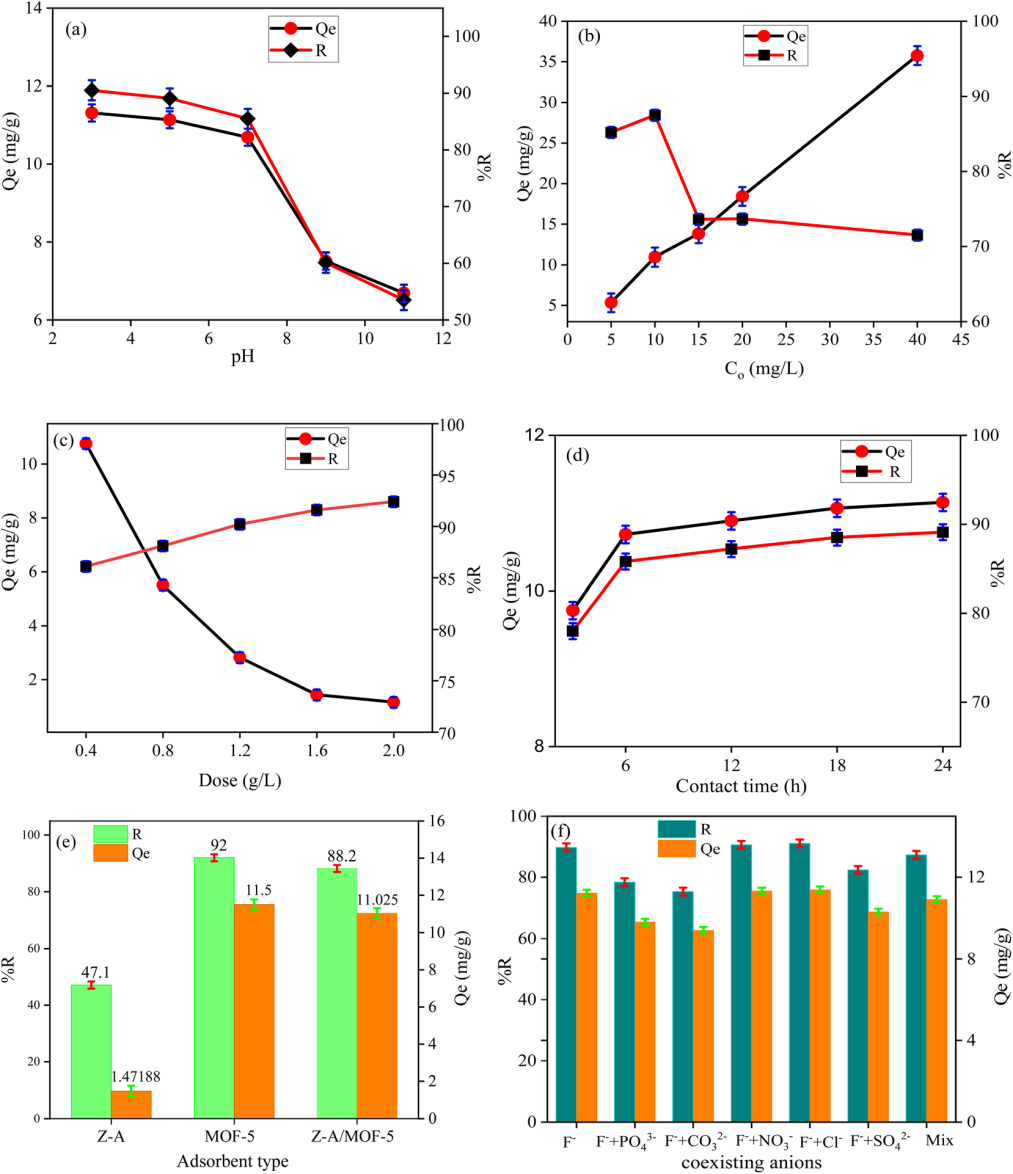
Effects of (a) pH value, (b) *C*_o_, (c) adsorbent dose, (d) contact time, (e) adsorbent type, and (f) co-existing anions on the defluoridation efficiency and capacity.

The defluoridation efficiency of the Z-A/MOF-5 composite was 90.50, 89.10, 85.50, 60.10, and 53.50% at pH of 3, 5, 7, 9, and 11, respectively (Fig. 8a). The maximum defluoridation capacity (14.61 mg g^−1^) was obtained at a pH of 3. Nevertheless, the acidic solution required an extra neutralization step for the defluoridation of F^−^ from groundwater.^7^ The defluoridation efficiency was maintained at a high level at pH 7 (10.68 mg g^−1^, 85.50%), which applies to the defluoridation of groundwater. Therefore, pH 7 was selected for further defluoridation studies. Below PZC, the surface of the adsorbent material is protonated and positively charged which enhances the defluoridation of F^−^ ion.^4^ At pH values beyond the PZC, the surface of the adsorbents is deprotonated (HO^−^ is formed), which results in low defluoridation capacity owing to electrostatic repulsion forces between the adsorbent's surface and F^−^.^19,51^ Above pH PZC (Fig. 7a), the progressive decrease of defluoridation efficiency is due to competition between OH^−^ and F^−^ for adsorption sites and coulombic repulsion between the negative surface of Z-A/MOF-5 and F^−^.^34,43^

The effect of F^−^*C*_o_ at F^−^ concentrations of 5, 10, 15, 20, and 40 mg L^−1^ resulted in 85.20, 87.50, 73.61, 72.25, and 68.46% defluoridation efficiency, respectively. The defluoridation efficiency decreased with the increased F^−^*C*_o_ (Fig. 8b). This could be the presence of high F^−^ in the solution which remains unabsorbed due to the saturation of the adsorbent sites.^2,12^ Conversely, the defluoridation capacity was 5.325, 10.94, 13.80, 18.41, and 35.75 mg g^−1^ at 5, 10, 15, 20, and 40 mg L^−1^ F^−^*C*_o_, which increased from 5.33 to 35.75 mg g^−1^ as the *C*_o_ of F^−^ ions increased from 5 to 40 mg L^−1^, respectively. As the *C*_o_ of F^−^ increased, the concentration gradient was created between adsorbent and adsorbate. This increased the driving force at the solid-liquid interface and there was a mass transfer of F^−^ into the adsorbent surface.^1,52^ Hence, the defluoridation capacity is remarkably increased with the increase of F^−^*C*_o_.

#### 3.2.3. Effect of adsorbent dose and contact time

The defluoridation efficiency of Z-A/MOF-5 adsorbent was 86.10, 88.10, 90.20, 91.60, and 92.40% at 0.4, 0.8, 1.2, 1.6, and 2.0 g L^−1^ of adsorbent dose, respectively. This is possibly due to the incremental increase of free available active sites on the adsorbent surface with higher adsorbent doses.^1,2^ Beyond the 1.2 g L^−1^ Z-A/MOF-5 dose, the defluoridation efficiency did not show a substantial increment due to the saturation of adsorbent sites.^16^ Thus, the 1.2 g L^−1^ Z-A/MOF-5 dose was selected for further defluoridation studies. Conversely, the defluoridation capacity was 10.76, 5.51, 2.82, 1.44, and 1.16 mg g^−1^ at 0.4, 0.8, 1.2, 1.6, and 2.0 g L^−1^ adsorbent doses, respectively. This is due to the accumulation of adsorbent particles together,^16^ which reduced the free active sites of adsorbent materials; this reduced the defluoridation capacity from 10.76 mg g^−1^ to 1.16 mg g^−1^ as the dose of Z-A/MOF-5 adsorbent increased from 0.4 to 2.0 g L^−1^ (Fig. 8c).

At contact times of 3, 6, 9, 12, and 15 h, the removal of F^−^ by Z-A/MOF-5 was 78.00.85.80, 87.20, 88.50, and 89.10%, respectively. Initially, the defluoridation rate increased and approached equilibrium at 6 h. Thus, 6 h was used as the optimal time for the defluoridation studies of groundwater. The defluoridation of F^−^ slowly increased with longer contact time, which confirms the heterogeneous nature of the as-synthesized Z-A/MOF-5 composite (Fig. 8d). The defluoridation capacity was 9.75, 10.73, 10.90, 11.06, and 11.14 mg g^−1^ at 3, 6, 9, 12, and 15 h adsorption time, respectively. Consequently, the contact time shows a positive effect on the defluoridation of drinking water.^2,9,10^

#### 3.2.4. Effects of adsorbent type and co-existing ions

The defluoridation of groundwater is highly influenced by the nature of adsorbents.^53^ Herein, the defluoridation efficiency of the as-synthesized adsorbents (Z-A, MOF-5, and Z-A/MOF-5) was 47.10, 92.00, and 88.20%, respectively (Fig. 8e). The defluoridation of F^−^ by Z-A could be associated with the presence of Al and Al–OH in the Z-A framework, which causes ligand exchange and formation of Al–F bonds.^52^ The other possible reason might be the presence of extra-framework (cations) in the Z-A framework.^2^ The 88.20% defluoridation efficiency of Z-A/MOF-5 is much greater than that of the pristine material Z-A (47.10%), indicating that the Z-A/MOF-5 composite has greater selectivity than Z-A. This could be due to the synergistic effect of the individual constituents in the Z-A/MOF-5 composite.^16^ This is why the intended work seeks to form a composite Z-A with MOF-5. Groundwater contains numerous co-existing anions (Table S1†), which may compete with F^−^ during the defluoridation processes.^1,52^ To investigate the impact of co-existing ions, the defluoridation studies were carried out using 10 mg L^−1^ salt solutions of NaCl, NaNO_3._, Na_2_CO_3_, Na_2_SO_4_, Na_2_HPO_3,_ and their combined form (Mix). The defluoridation efficiency for without ions (F^−^), PO_4_^3−^, CO_3_^2−^, NO_3_^−^, Cl^−^, SO_4_^2−^, and their mix form was 89.80, 78.40, 75.30, 90.60, 91.10, 82.40, and 87.30%, respectively. The defluoridation efficiency decreases with the addition of Na_2_CO_3,_ Na_2_SO_4_, and Na_2_HPO_4_ (Fig. 8f). The final pH of the F^−^ solution changed from 7 to 6.9, 7.1, 11.2, 7.6, and 8.8 upon the addition of NaCl, NaNO_3,_ Na_2_CO_3_, Na_2_SO_4_, and Na_2_HPO_4_, respectively. This alters the surface charges of the synthesized material (Fig. 7b). This reduction in efficiency could be due to the competition of OH^−^ and F^−^ for the adsorbent sites.^1,43^ The other possible reason is the high surface charge densities and larger ionic radii of PO_4_^3−^ (0.238 nm) and SO_4_^2−^ (0.230 nm) than that of the F^−^ (0.133 nm) anions; high surface charge densities and larger ionic radii preferentially adsorb on the surface of synthesized adsorbents more than F^−^.^1,51^ A complementary result was reported by Raghav *et al.*^51^ PO_4_^3−^ also could form complexes with adsorbents by inner and outer sphere mechanisms and better competed with F^−^ ions and reduced the defluoridation of F^−^ from groundwater.^9,16^ In a nutshell, co-existing PO_4_^3−^, SO_4_^2−^ and CO_3_^2−^ show a negative effect on the removal of F^−^ while Cl^−^ and NO_3_^−^ show a slight positive effect^3^ (Fig. 8f). Overall, their combined form (Mix) does not show an ampere effect on the defluoridation efficiency of Z-A/MOF-5 composite. Consequently, the synthesized adsorbent Z-A/MOF-5 can adsorb F^−^ from groundwater containing a complex matrix of co-existing ions.

### 3.3. Kinetic studies

**Fig. 9:**
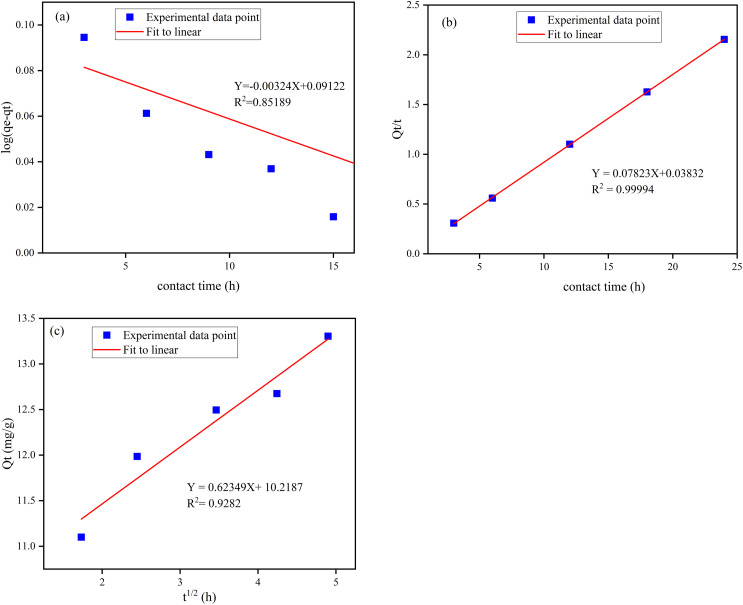
(a) Pseudo-first-order model, (b) pseudo-second-order model, and (c) intraparticle diffusion model.

The kinetic properties of the adsorption process can be explained through intraparticle diffusion, pseudo-second, and pseudo-first-order models.^10,16^ The linear plot of *t vs.* log(*Q*_e_ − *Q*_*t*_), and *t vs. t*/*Q*_*t*_ were pseudo-first-order and pseudo-second-order, respectively (Fig. 9a and b). The best-fitting model was selected based on the correlation coefficient (*R*^2^), and the conformity between experimental data (*Q*_m. exp_) and model-predicted values (*Q*_m. Fit_). The correlation coefficients (*R*^2^) of the pseudo-first and pseudo-second-order were 0.852 and 0.999, respectively. The calculated adsorption capacity using pseudo-second-order and pseudo-first equations were 11.33 and 1.23 mg g^−1^, respectively (Table S2†). Accordingly, the defluoridation of F^−^ on the Z-A/MOF-5 composite fits the pseudo-second-order kinetics, which implies that the defluoridation mainly takes place *via* chemosorption.^9^ The plots of the amount of F^−^ adsorbed per unit mass of adsorbents (*Q*_*t*_) and the square root of time (*t*^1/2^) are used to show the intraparticle diffusion of F^−^ on the Z-A/MOF-5 adsorbent.^16^ The intraparticle diffusion rate and boundary layer were found to be 0.624 mg g^−1^ min^−1^ and 10.22, respectively; the particles are diffusing though a high resistance adsorbent's surface at 0.624 mg g^−1^ min^−1^ diffusion rate. The defluoridation of F^−^ partially proceeds by diffusion.^1^ This suggests that the adsorption is governed by the intraparticle diffusion model.^16^ Besides, the linear curves do not pass through the origin (Fig. 9c), which implies that the defluoridation mechanism of F^−^ on Z-A/MOF-5 adsorbent proceeds through the surface adsorption and intraparticle diffusion method.^1,16^

### 3.4. Adsorption isotherms

**Fig. 10:**
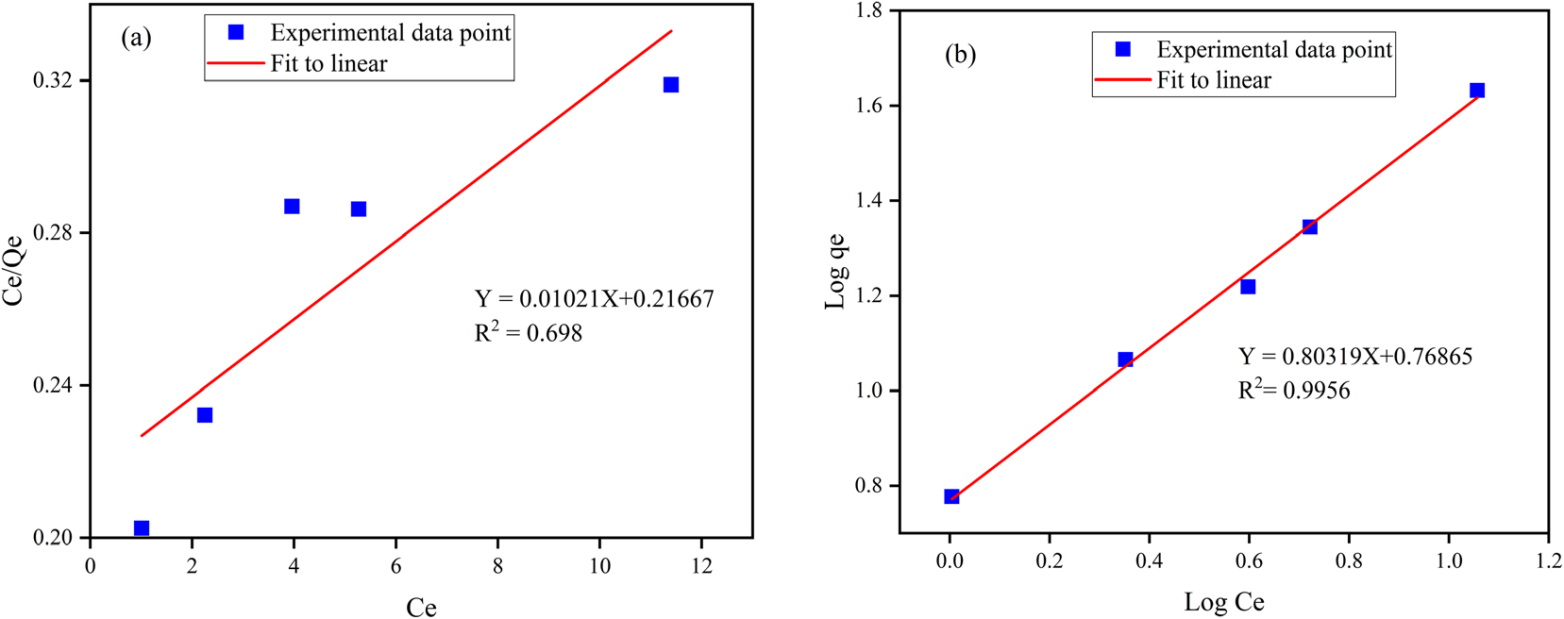
(a) Langmuir adsorption isotherm model and (b) Freundlich adsorption isotherm model.

The Freundlich and Langmuir isotherm model^9^ of the synthesized Z-A/MOF-5 adsorbent was studied (Fig. 10a and b) to confirm the heterogeneous and homogeneous nature, respectively. The values of *Q*_m_ and *K*_L_ of the Langmuir adsorption parameters were 97.94 mg g^−1^ and 0.0472 L mg^−1^, respectively (Table S3†). The calculated value of *Q*_m_ (97.94 mg g^−1^) is quite far from the experimental value (10.725 mg g^−1^). Besides, the Langmuir isotherm model shows a lower *R*^2^ value (0.83782) than Freundlich (0.9956). Thus, the Langmuir isotherm model is not appropriate to describe the adsorption behavior of F^−^ on the Z-A/MOF-5 surface. In the Freundlich model, the values of *n* and 1/*n* were 1.18005 (*n* > 1) and 0.84742 (0 < 1/*n* < 1), respectively which implies that the defluoridation is sympathetic.^51^ Consequently, the defluoridation of F^−^*via* Z-A/MOF-5 best fits the Freundlich models, indicating that the defluoridation of F^−^ occurred on the heterogeneous surface of the Z-A/MOF-5 adsorbent.

### 3.5. Response surface method (RSM) study

The mutual interaction impacts of defluoridation parameters (contact time, *C*_o_ of F^−^, and adsorbent dose) were also utilized at 17 runs using the Box–Behnken model (Table S4†) at pH = 7.^54,55^ Hereafter, the response and the input variables are expressed using a quadratic model-coded eqn (10). The positive or negative term indicates the synergistic or antagonistic effect of the term, respectively.^51^ The coefficients of each variable are 2.00, 1.48, and −5.11, confirming that *A* and *B* have a positive effect, whilst *C* has a negative effect on the removal of F^−^ by Z-A/MOF-5 adsorbent.

10 % *R* = 85.89 + 2.00*A* + 1.48*B* − 5.11*C* − 0.0425*AB* + 0.12*AC* − 0.3125*BC* − 1.07*A*^2^ − 0.3375*B*^2^ − 3.06*C*^2^

where, *A*, *B*, and *C* are the coded values of the operation variables adsorbent dose, *C*_o_ of F^−^, and contact time, respectively.

The model fitness was checked based on the values of the coefficient of determination (*R*^2^). The proximity of the *R*^2^ value to unity (*R*^2^ = 0.9988) and the intimacy of the adjusted *R*^2^ (*R*_adj_^2^ = 0.9973) and predicted *R*^2^ (*R*_pred_^2^ = 0.9812) values to each other show differences <0.2.^53,54^ The predicted *R*^2^ value reasonably agreed with the adjusted *R*^2^ value, which approves the model's fitness (Table 1). Besides, the precision that measures the signal-to-noise ratio (AP = 85.5225) is greater than 4, indicating a good signal and accurate model fit.^56^ The model's statistical significance is proven by its high *F*-value (662.35) and low *p*-value (<0.0001), respectively.^51^ According to the data presented above, there is only a 0.01% probability that the *F*-value of this magnitude will occur owing to noise. In this regard, *A*, *B*, *C*, *AB*, *AC*, and *A*^2^ are significant (Table 1). Consequently, the Box–Behnken model was verified and statistically proved to be reliable and adequate for the defluoridation of drinking water.^53^

**Table 1:** Analysis of variance of the ANOVA for the response surface quadratic model

Source	Sum of squares	df	Mean square	*F*-value	*p*-value	Remarks
Model	306.21	9	34.02	662.35	<0.0001	Significant
*A*-dose	31.96	1	31.96	622.18	<0.0001	Significant
*B*-time	17.41	1	17.41	338.83	<0.0001	Significant
*C*–*C*_o_	209.20	1	209.20	4072.65	<0.0001	Significant
*AB*	0.0072	1	0.0072	0.1407	0.7187	Insignificant
*AC*	0.0576	1	0.0576	1.12	0.3248	Insignificant
*BC*	0.3906	1	0.3906	7.60	0.0282	Significant
*A* ^2^	4.82	1	4.82	93.85	<0.0001	Significant
*B* ^2^	0.4796	1	0.4796	9.34	0.0184	Significant
*C* ^2^	39.43	1	39.43	767.52	<0.0001	Significant
Residual	0.3596	7	0.0514			
Lack of fit	0.3596	3	0.1199			
Pure error	0.0000	4	0.0000			
Cor total	306.57	16				
Std. Dev.	0.2266			Adjusted *R*^2^	0.9973	
Mean	83.79			Predicted *R*^2^	0.9812	
C.V.%	0.2705			Adeq precision	85.5225	

#### 3.5.1. Mutual interaction effects

**Fig. 11:**
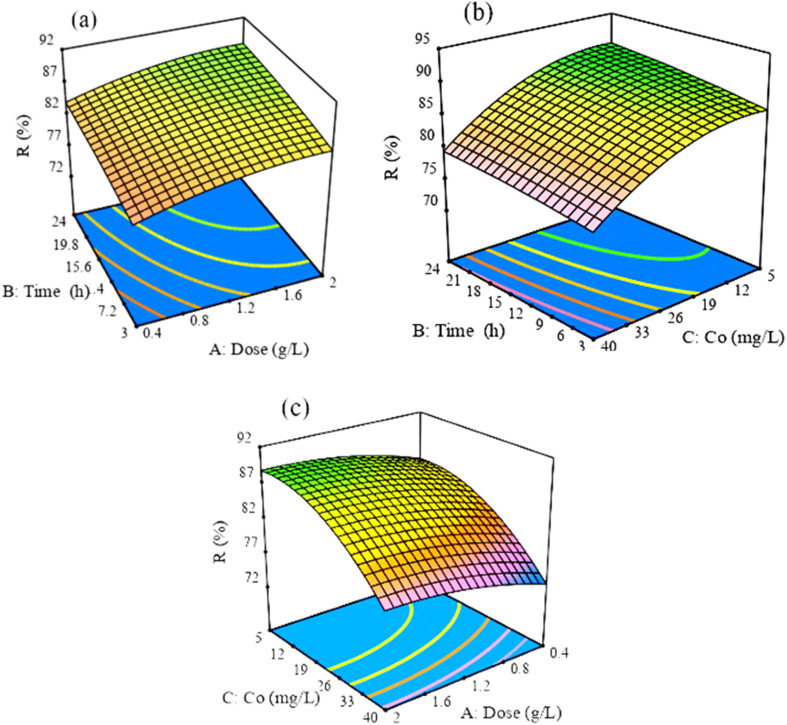
Surface response plots of (a) contact time and adsorbent dose, (b) contact time and *C*_o_, and (c) adsorbent dose and *C*_o_.

The mutual interaction impacts of Z-A/MOF-5 dose and contact time on the removal of F^−^ showed positive effects (Fig. 11a). As the Z-A/MOF-5 dose increases, there are free available active sites, which can adsorb more F^−^.^51^ In the same manner, the adsorbates have more chances to be adsorbed as the contact time increases. Accordingly, both the Z-A/MOF-5 dose and contact time show a positive effect. The mutual impact of contact time and *C*_o_ of F^−^ exhibits a negative impact on the defluoridation efficiency of the Z-A/MOF-5 composite (Fig. 11b). Individually, the *C*_o_ of F shows negative effects, whereas the contact time shows a positive impact on the removal of F^−^.^53,57^ The collaboration of adsorbent dose and *C*_o_ of F^−^ (Fig. 11c) also shows a negative effect on the defluoridation of F^−^.

### 3.6. Adsorption mechanisms

**Fig. 12:**
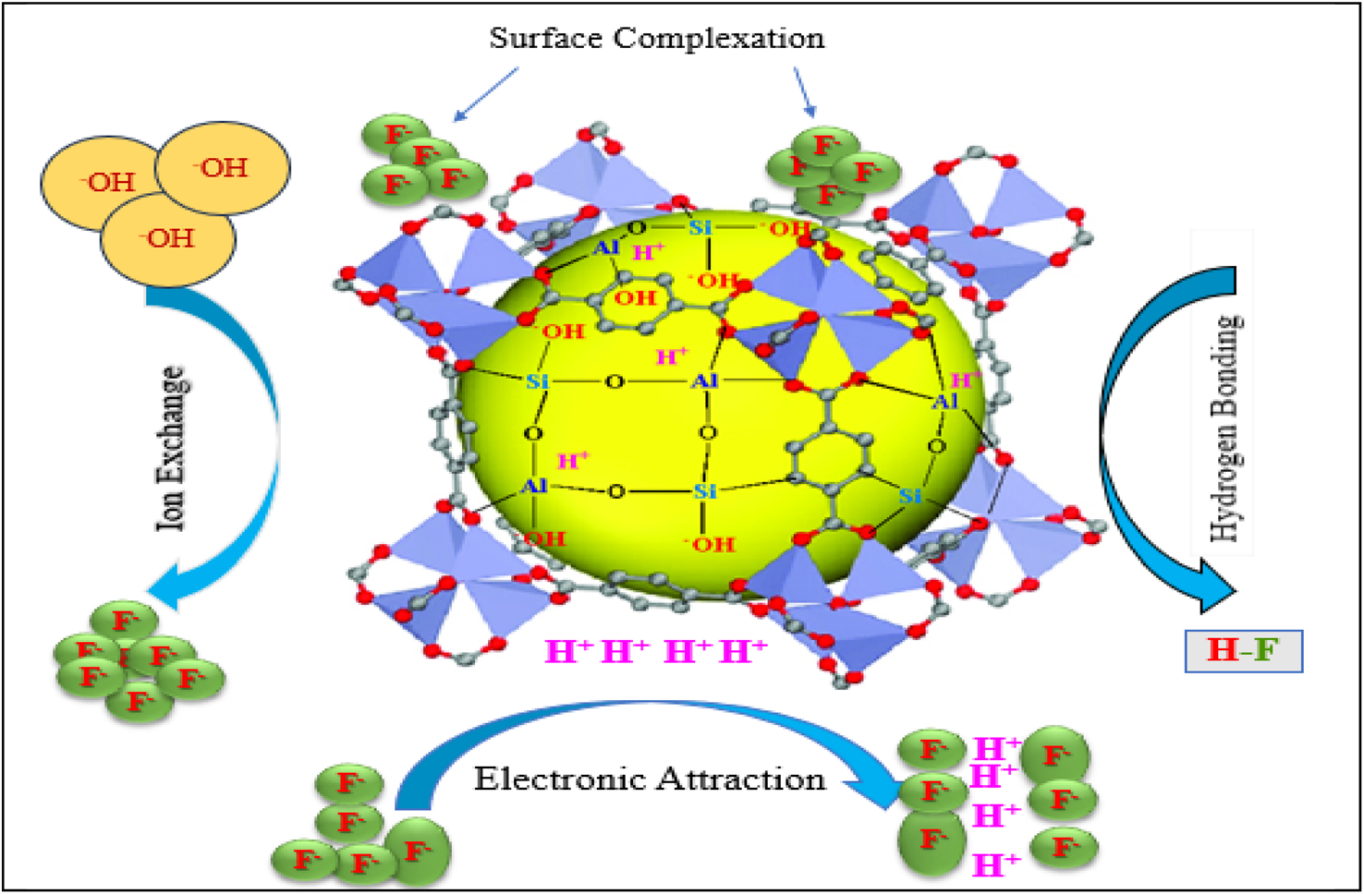
Defluoridation mechanism of F^−^ on the Z-A/MOF-5 surface.

The defluoridation mechanism of F^−^ on the Z-A/MOF-5 surface could proceed through surface complexation,^52^ ion exchange,^43^ hydrogen bonding,^51^ ligand exchange,^9^ and electrostatic interaction^1^ (Fig. 12). Due to isoelectronic and comparable size of the hydroxyl groups on the metal nodes within the structure of Z-A/MOF-5, the hydroxyl groups can be substituted with F^−^ and the defluoridation of F^−^ proceeds through anion exchange.^16,17,52^ This could be due to the displacement of M–OH bonds and the formation of M–F bonds (M = Al, Zn, Si) in the Z-A/MOF-5 framework. Loss of the OH vibration bands from 3200 to 3500 cm^−1^ (Fig. 3d) suggests the replacement of the hydroxyl group by F^−^ through ion exchange or hydrogen bonds (F⋯H–O).^34^ Most importantly, the defluoridation mechanism occurred *via* electrostatic interactions. According to PZC (Fig. 7), the surface of the as-synthesized adsorbents is protonated and positively charged up to a pH of 8.1, which is accessible for the removed F^−^*via* electrostatic interactions. In the Z-A/MOF-5 framework, the Zn^2+^ metal center could be replaced with Al^3+^ to form the Al-MOF-5 extra framework, which can remove F^−^ from groundwater through electrostatic interactions.^43^ In this context, the hard acid Al^3+^ preferably interacted with the hard F^−^ base through electrostatic interactions.^8^

### 3.7. Real sample analysis and recyclability test

**Fig. 13:**
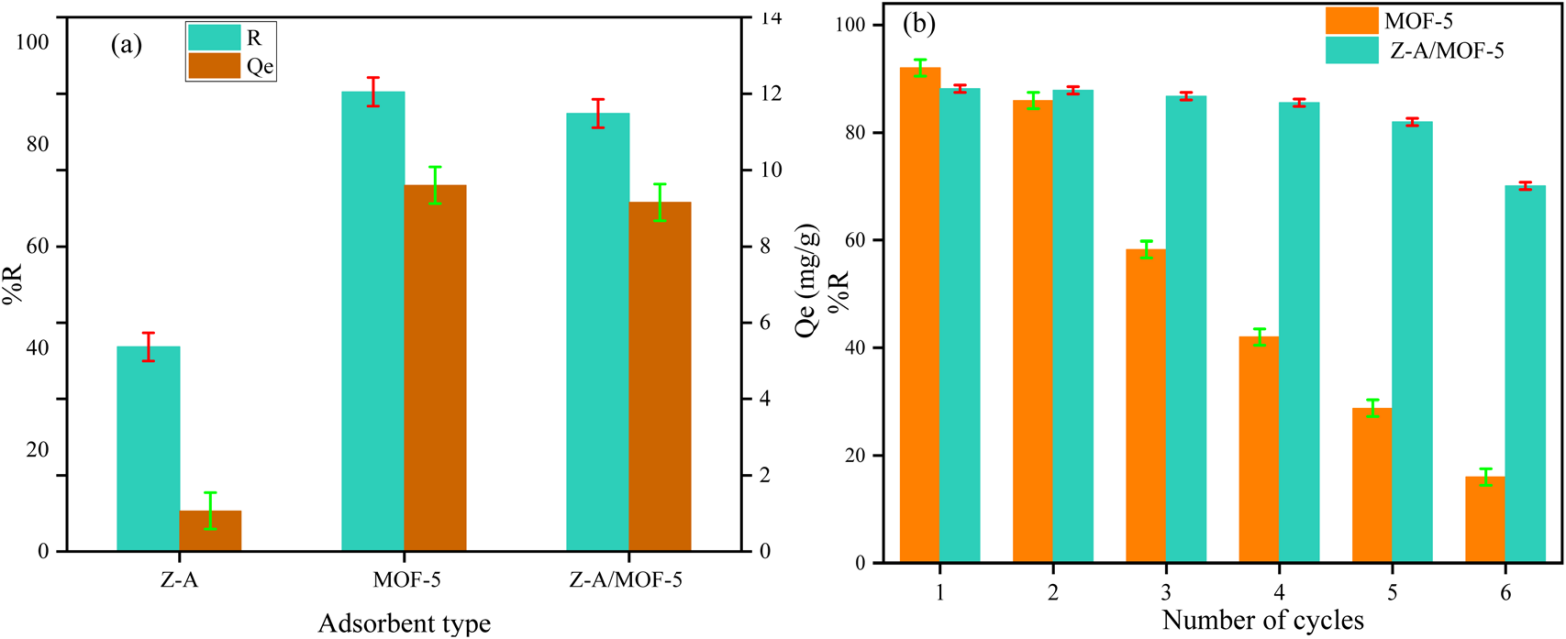
(a) Defluoridation of real sample analysis and (b) recyclability test (adsorbent dose = 1.2 g L^−1^, *C*_o_ = 8.5 mg L^−1^, contact time = 6 h, and pH = 6.8).

Before applying the defluoridation test, the content of groundwater particularly anions (Cl^−^, NO_3_^−^, CO_3_^2−^, SO_4_^2−^, and PO_4_^3−^) was analyzed using the standard method^1,58^ and the results were shown in Table S1.† Then, the performance of the as-synthesized adsorbent was applied for the defluoridation of real water samples containing 12.25 and 8.5 mg L^−1^ F^−^*C*_o_ which were taken from Ziway and Kenteri town, Ethiopia. The equilibrium concentration of F^−^ was reduced to 1.48 and 0.82 mg L^−1^, respectively, which meets the permissible limit of F^−^ concentration in portable water recommended by the WHO. Accordingly, the as-synthesized Z-A/MOF-5 composite was used for the removal of F− from drinking water. Furthermore, the defluoridation efficiency of Z-A, MOF-5, and Z-A/MOF-5 using 8.5 mg L^−1^ F^−^*C*_o_ was 40.24, 90.35, and 86.12%, respectively (Fig. 13a). The defluoridation efficiency of the as-synthesized MOF-5 was 92.00, 86.00, 58.30, 42.00, 28.80, and 16.00% for the 1st, 2^nd^, 3^rd^, 4^th^, 5^th^, and 6^th^ runs, respectively (Fig. 13b). Therefore, the recyclability of MOF-5 abruptly decreased from the 1st to the 6th cycles. Interestingly, the as-synthesized Z-A/MOF-5 composite shows 88.20%, 87.90%, 86.80%, 85.60, 82.00%, and 70.10% defluoridation efficiencies for the 1^st^, 2^nd^, 3^rd^, 4^th^, 5^th^, and 6^th^ runs, respectively (Fig. 13b).

### 3.8. Comparison of Z-A/MOF-5 defluoridation efficiency with previous reports

The defluoridation efficiency (*R*) and capacity (*Q*_e_) of the as-synthesized Z-A/MOF-5 composite were compared with other adsorbents, which were reported previously (Table 2). The as-synthesized adsorbent shows good defluoridation efficiency and capacity even at low adsorbent doses. Therefore, the Z-A/MOF-5 composite is a good adsorbent for the defluoridation of groundwater.

**Table 2:** Comparison of Z-A/MOF-5 defluoridation efficiency with previous reports

S.no.	Adsorbents	pH	Dose (g L^−1^)	Time (h)	*C*_o_ (mg L^−1^)	*R* (%)	*Q*_e_ (mg g^−1^)	Ref.
1	Zeolite/cationic surfactants	5.5.	5.0	24	5	88.40	5.5	11
2	Zirconium-MOF (MOF-801)	—	1	2	10	92.30	19.42	59
3	Al hydroxide-loaded zeolite (AHZ)	6.1	8	2	10	92.00	8.12	34
4	Alum-modified zeolite (Alum-Z)	6	6	2	5	98.87	2.43	17
5	Z-A/MOF-5	3	1.2	6	10	88.20	11.025	This study
6	Z-A/MOF-5	*7*	1.2	6	10	85.50	10.68	This study

## 4. Conclusions

Z-A, MOF-5, and their composite (Z-A/MOF-5) adsorbents were synthesized by the solvothermal method for the defluoridation of F^−^ from groundwater. The phase structure, functional groups, thermal stability, elemental composition, and morphology of the synthesized adsorbents were also characterized using PXRD, FT-IR, TGA, and SEM-EDX to confirm the formation of adsorbent materials. The defluoridation efficiency of Z-A/MOF-5 adsorbent was started by optimizing the pH, adsorbent dose, *C*_o_, and contact time. The maximum defluoridation efficiency (88.20%) and capacity (11.025 mg g^−1^) were obtained at pH 3, adsorbent dose of 1.2 g L^−1^, contact time of 6 h, and F^−^*C*_o_ of 10 mg L^−1^. However, the defluoridation efficiency does not show a substantial decrement up to a pH of 7; thus, the adsorbent is applicable at a wide pH range for the defluoridation of groundwater. The Box–Behnken design model with the three independent variables (contact time, *C*_o_ of F^−^, and adsorbent dose) was detected to present their interaction effects. The Z-A/MOF-5 dose and contact time showed a positive effect while the Z-A/MOF-5 dose and F^−^*C*_o_, and contact time and F^−^*C*_o_ showed negative effects on the removal of F. The Freundlich adsorption isotherm model and pseudo-second-order kinetics were well-fitted to explain the defluoridation process. Interestingly, the recyclability study resulted in 88.20%, 87.90%, 86.80%, 85.60%, 82.00%, and 70.10% defluoridation efficiency for the 1^st^, 2^nd^, 3^rd^, 4^th^, 5^th^, and 6^th^ sequential runs, respectively. This suggests that the Z-A/MOF-5 composite is effective and can be reused for the defluoridation of groundwater. The application of the as-synthesized adsorbent for the defluoridation of real water samples containing 12.25 and 8.5 mg L^−1^ F^−^*C*_o_ reduced [F^−^] to 1.48 and 0.82 mg L^−1^, respectively which meets the permissible limit of F^−^ concentration in drinking water recommended by the WHO. Thus, the synthesized adsorbent is useful for the community and a good candidate for the defluoridation of groundwater.

## Data availability

The data supporting this article have been included as part of the ESI data.†

## Author contributions

T. D.: methodology, data analysis, characterized and wrote the full manuscript. E. A.: supervising, conceptualization, and editing of the manuscript. T. J.: revision, methodology, and resources. All authors have read and reached an agreement for the publication of the manuscript.

## Conflicts of interest

There are no conflicts to declare.

## Supplementary Material

RA-015-D5RA01995H-s001
