# Supplementary material for: Synthesis of a zeolite-a/MOF-5 composite for the defluoridation of groundwater
Source: RSC Adv. 2025 May 9;15(19):15200–17. doi: 10.1039/d5ra01995h (PMC12062793; doi:10.1039/d5ra01995h)
Supplement: RA-015-D5RA01995H-s001 [file RA-015-D5RA01995H-s001.pdf]

Supplementary Information (SI) for RSC Advances.
This journal is © The Royal Society of Chemistry 2025

Supplementary Information

Synthesis of Zeolite-A/MOF-5 Composite for the Defluoridation of Groundwater

Tessema Derbe[abc*], Taju Sani[ab], and Enyew Amare Zereffa[d]

[a] Department of Industrial Chemistry, Addis Ababa Science and Technology University, P.O. Box 16417, Addis Ababa, Ethiopia

[b] Nanotechnology Center of Excellence, Addis Ababa Science and Technology University, P.O. Box 1647, Addis Ababa, Ethiopia

[c] Department of Chemistry, Wachemo University, P.O. Box 667, Hossana, Ethiopia

[d] Department of Applied Chemistry, School of Applied Natural Science, Adama Science and Technology University, P.O. Box 1888, Adama, Ethiopia

* Correspondence: benyamderbe@gmail.com: +251-9-12-97-33-96

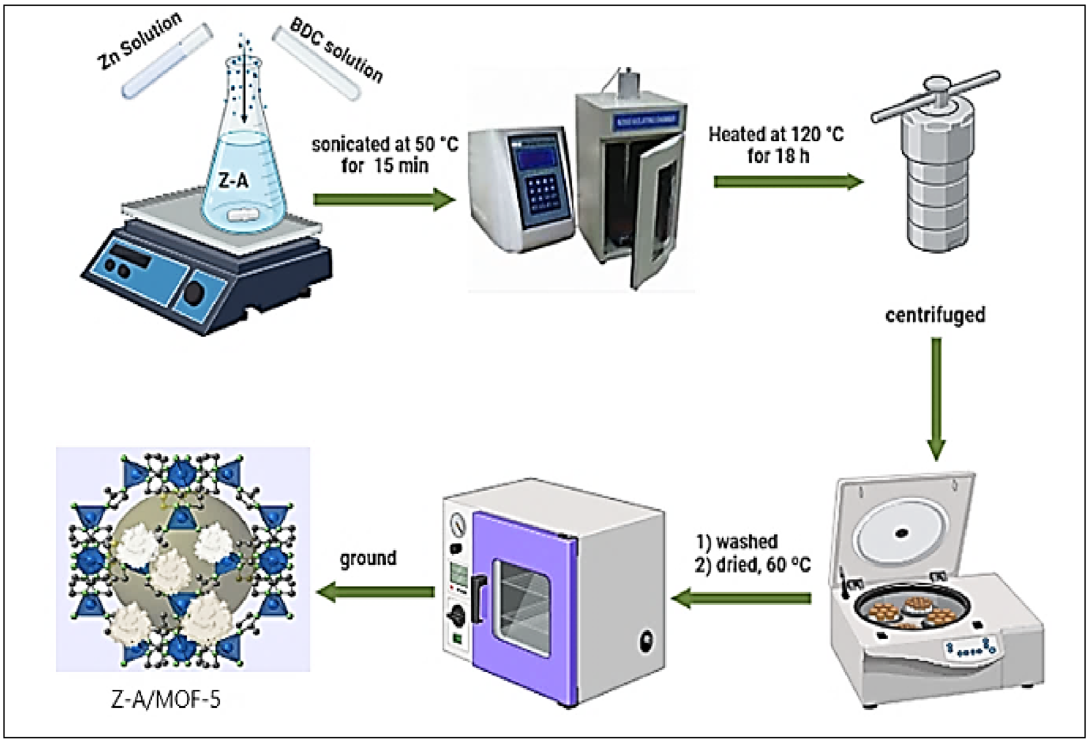

Fig. S1 Synthesis of Z-A/MOF-5 composite by solvothermal method.

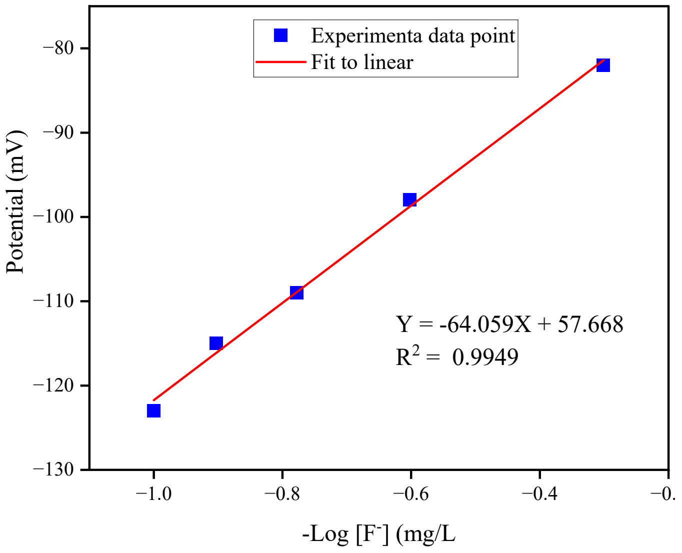

Fig. S2 Calibration curve of F^- standard solutions.

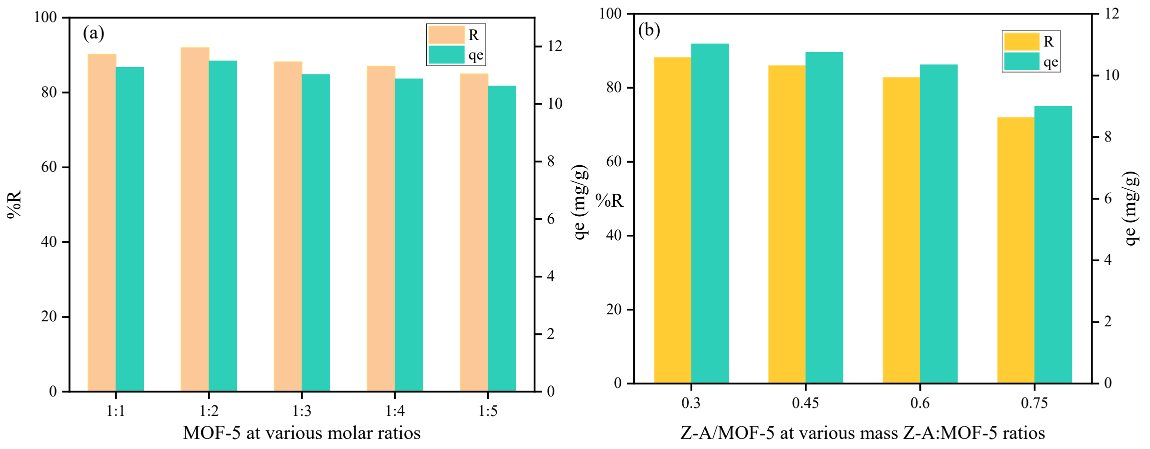

Fig. S3 Preliminary defluoridation efficiency and capacity of synthesized (a) MOF-5 and (b) Z-A/MOF-5 adsorbents.

Table S1 Physicochemical properties of groundwater samples were collected from Ziway, and Kenteri town, Ethiopia.

S. No	Parameter	Before adsorption		After adsorption	
		Ziway	Kenteri	Ziway	Kenteri
1	SO_4^{2-} (mg/L)	5.00	4.80	3.10	2.52
2	pH	6.8	7.5	7.8	7.6
3	NH_3 (mg/L)	0.67	1.54	0.62	1.21
4	NO_3^- (mg/L)	1.90	2.82	1.68	2.45
5	Cl^- (mg/L)	337.00	136.74	141.00	127.86
6	F^- (mg/L)	8.50	12.25	0.57	0.99
7	PO_4^{3-} (mg/L)	4.15	5.94	2.89	4.00
8	CO^-_3 (mg/L)	1.29	1.40	0.54	0.35

Table S2 Result for Pseudo-first-order and pseudo-second-order model

Pseudo-first-order			Pseudo-second-order			
Qm. fit (mg/g)	K_1 (hr^{-1})	R^2	Qm. fit (mg/g)	Qm. exp(mg/g)	R^2	K_2 (g $mg^{-1}hr^{-1}$)
1.23373	0.00229	0.85189	11.334	10.725	0.9994	0.203145

Table S3 Result for Langmuir isotherm and Freundlich adsorption isotherm model

Langmuir model			Freundlich model		
Qm (mg/g)	K_L(L/mg)	R^2	K_F	1/n	R^2
97.943	0.047146	0.83782	5.87016	0.84742	0.9956

Table S4 A number of runs applied using the Box-Behnken quadratic model in RSM

		Factor 1	Factor 2	Factor 3	Response 1
Std	Run	A:Dose	B:Time	C:Co	R
		g/L	h	mg/L	%
2	1	2	3	22.5	84.98
11	2	1.2	3	40	76.07
17	3	1.2	13.5	22.5	85.89
3	4	0.4	24	22.5	84.07
12	5	1.2	24	40	78.28
8	6	2	13.5	40	78.98
9	7	1.2	3	5	86.08
6	8	2	13.5	5	88.56
14	9	1.2	13.5	22.5	85.89
7	10	0.4	13.5	40	74.72
13	11	1.2	13.5	22.5	85.89
1	12	0.4	3	22.5	80.92
10	13	1.2	24	5	89.54
5	14	0.4	13.5	5	84.78
4	15	2	24	22.5	87.96
16	16	1.2	13.5	22.5	85.89
15	17	1.2	13.5	22.5	85.89
